# A Smartphone-Based Implicit Theories Intervention for Health Behavior Change: Randomized Trial

**DOI:** 10.2196/36578

**Published:** 2023-06-15

**Authors:** Mike Schreiber, Simone Dohle

**Affiliations:** 1 Faculty of Psychology University of Vienna Vienna Austria; 2 Social Cognition Center Cologne Department of Psychology University of Cologne Cologne Germany; 3 Institute of General Practice and Family Medicine University Hospital Bonn University of Bonn Bonn Germany

**Keywords:** daily diary, ecological momentary assessment, health behavior, implicit theories, lay theories, mindsets, multiple health behavior change, randomized trial, smartphone-based intervention

## Abstract

**Background:**

Implicit theories of health describe individuals’ beliefs about the malleability of health. Individuals with an incremental theory of health believe that health, in general, is malleable, whereas individuals with an entity theory of health endorse the idea that health is largely fixed and predetermined. Previous research has shown that an incremental theory of health is associated with beneficial health outcomes and behaviors. A mobile health implicit theories intervention could be an effective way to increase health-promoting behaviors in the general population.

**Objective:**

The aim of this study was to estimate the effect of a smartphone-based intervention designed to promote an incremental theory of health on the frequency of health-promoting behaviors in everyday life. The study used ecological momentary assessment to measure health behavior change.

**Methods:**

This 2-arm, single-blind, delayed intervention design included 149 German participants (mean age 30.58, SD 9.71 years; n=79 female). Participants were asked to report their engagement in 10 health-promoting behaviors throughout the day for 3 weeks. Participants were randomly assigned to either an early intervention group (n=72) or a delayed intervention group (n=77). The intervention materials, designed to promote an incremental theory of health, were provided to participants after 1 week (early intervention group) or 2 weeks (delayed intervention group) of baseline behavior measurement. Data for this study were collected between September 2019 and October 2019.

**Results:**

A paired-samples 2-tailed *t* test revealed that participants reported a stronger incremental theory after responding to the intervention materials (mean 5.58, SE 0.07) compared with incremental theory measured in an entry questionnaire (mean 5.29, SE 0.08; *t*_148_=4.07, SE 0.07; *P*<.001; 95% CI 0.15-0.43; *d*=0.33). Multilevel analyses showed that participants reported engaging in health-promoting behaviors more often after being presented with the intervention materials compared with baseline across conditions (*b*=0.14; *t*_146.65_=2.06, SE 0.07; *P*=.04; 95% CI 0.01-0.28). However, when the analysis was conducted separately for the early and delayed intervention groups, the intervention effect was only significant for the delayed intervention group (*b*=0.27; *t*_1492.37_=3.50, SE 0.08; *P*<.001; 95% CI 0.12-0.42). There was no significant increase in health-promoting behaviors for the early intervention group (*b*=0.02; *t*_69.23_=0.14, SE 0.11;*P*=.89; 95% CI −0.2 to 0.23).

**Conclusions:**

This study suggests that a smartphone-based intervention designed to promote an incremental theory of health is a cost- and time-effective approach to increase the frequency of engaging in health-promoting behaviors. However, research is needed to understand the reasons for the difference in intervention effects between the early and delayed intervention groups. The results of this study can guide the development of future digital health interventions that focus on implicit theories to promote health behavior change.

**Trial Registration:**

DRKS – German Clinical Trials Register DRKS00017379; https://drks.de/search/de/trial/DRKS00017379

## Introduction

### Background

According to the World Health Organization [1], 71% of all worldwide deaths are attributed to noncommunicable diseases like cardiovascular diseases, cancer, respiratory diseases, or diabetes. The risk of having such a disease can decrease by adopting a healthier lifestyle that includes sufficient physical activity, a healthy diet, and avoiding harmful substances like tobacco or alcohol [1,2]. Engagement in such health-promoting behaviors often involves a high level of self-regulatory strategies [3,4]. An essential prerequisite for successful self-regulatory processes is *implicit theories* [5]. Implicit theories (sometimes also framed as mindsets or lay theories) refer to people’s beliefs about the changeability of human attributes and characteristics [5,6]. According to Dweck’s [6] framework, people differ in the extent to which they hold an *incremental theory*, that is, assuming that a given attribute is developable and malleable, versus an *entity theory*, that is, assuming that an attribute is fixed and stable. Recent research shows that a stronger incremental theory of health has a positive influence on maintaining a healthy lifestyle across multiple health behavior domains [7-10]. Extending these findings, the main aim of this randomized trial was to investigate whether promoting an incremental theory of health increases the frequency of performing health-promoting behaviors in daily life.

### Implicit Theories

Early research about implicit theories mainly focused on assumptions about the changeability of intelligence [11,12] or personality [13]. Since this first research, implicit theories have been studied across a wide array of domains like willpower [14,15], morality [16], stereotypes [17], and interpersonal relationships [18]. The majority of studies found that holding a stronger incremental theory in one domain (ie, assuming that the given characteristic is malleable) leads to positive outcomes [5,19]. For example, in a meta-analysis across 113 studies, holding an incremental theory was found to predict successful goal setting, goal monitoring, and goal operating, and, in turn, better self-regulation [5]. Therefore, researchers have developed many interventions to foster an incremental theory to create positive changes for individuals. The modes of delivering such interventions range from single-session approaches [20,21] to multisession approaches [12,22] and large-scale educational programs (eg, the Project for Educational Research That Scales [23]).

In the past decade, research about implicit theories has also become popular in different health domains, like weight management [22,24-26], physical activity [27,28], smoking [29,30], addiction [31,32], and mental health [21,33]. For example, it has been shown that an incremental theory can protect against setback-related weight gain [22], is related to higher motivation and intention to achieve a healthy weight [26], leads to greater motivation to quit smoking [31], and decreases anxiety and depressive symptoms [21].

Implicit theories in different domains are not necessarily interconnected [6,34]. For example, one might believe that one’s body weight is rather fixed around a given set point while also thinking that smoking behavior can be changed easily. Therefore, implicit theories have not only been studied in single health domains but also for health in general. Such generalized implicit theories have been examined concerning their impact on multiple health behavior domains [7-10]. In that sense, an incremental theory of (general) health regards the assumption that health is malleable and changeable, whereas an entity theory of health implies that health is perceived as fixed and stable [7-9]. Correlational research has shown that holding an incremental theory of health is related to performing health-promoting [7] and health-protective behaviors [10]. In addition, experimental findings suggest that a strengthened incremental theory of health leads to more positive attitudes toward different health-promoting behaviors [7], stronger intentions to eat healthily [8], and healthier food choices [7]. Previous research is therefore limited. Although correlative research shows evidence for the importance of an incremental theory of health for a healthy lifestyle, it cannot be interpreted causally. Existing experimental studies, on the other hand, generally focus only on one health behavior (eg, eating behavior). Therefore, the main purpose of this study is to investigate whether an intervention that promotes an incremental theory of health influences a variety of health-promoting behaviors. Compared with an intervention that focuses only on implicit theories in a single health domain (eg, body weight or physical activity), focusing on implicit theories of general health may serve as an efficient strategy to encourage multiple health behavior change. In contrast to stationary settings, the delivery of this intervention via digital technologies offers the opportunity to reach a larger audience in a sustainable manner while minimizing implementation costs [35,36]. To increase ecological validity and to minimize recall and retrieval bias, engagement in health-promoting behaviors is measured using ecological momentary assessment in the form of daily diaries [37]. Similarly, it has been shown that stronger incremental theories of health were connected to a higher frequency of performing health-promoting behaviors in daily life measured using experience sampling (study 4) [7]. As these results were only correlational, the present research aims to provide causal insights into whether an intervention to foster incremental theories increases health behaviors in daily life. Therefore, we make the following hypothesis: Being confronted with a smartphone-based intervention to foster incremental theories of health increases the frequency of performing health-promoting behaviors in daily life.

## Methods

### Study Design

We conducted a 2-arm, randomized trial to investigate whether fostering an incremental theory of health increases the frequency of performing health-promoting behaviors in daily life. The intervention was conceptualized as a delayed-start design [38,39], in which both groups received intervention material at different times. The intervention was delivered via participants’ smartphones using Qualtrics (Qualtrics International) questionnaires and included that participants kept a daily diary for 3 weeks. Participants were randomly assigned (single-blind) to an early or delayed intervention group using Qualtrics’ randomizer while maintaining an evenly distributed number of participants in each group (1:1 block randomization).

At the beginning of the study (day 0), all participants responded to an entry questionnaire to measure implicit theories of health, health locus of control, health-related self-efficacy, health-related outcome expectancy, health status, health value, health change motivation, and anthropometric (height and weight) and demographic variables (age, gender, education, and occupation). One day after responding to the entry questionnaire, the daily diary phase started. Over the course of 3 weeks (21 days), participants received daily invitations to complete a short questionnaire via texting distributed via SurveySignal [40]. Participants received the invitations daily at 8 PM and had to respond within 4 hours. In these daily questionnaires, participants were asked to indicate whether they performed 10 different health-promoting behaviors throughout the respective day. The number of daily performed health-promoting behaviors served as primary outcome measure. Depending on the assigned condition, participants received intervention materials to foster an incremental theory either after 7 (early intervention group) or 14 days (delayed intervention group) of baseline behavior measurement. After 21 days, we invited participants to participate in a follow-up questionnaire measuring the same constructs—except anthropometric and demographic items—as in the entry questionnaire. Table 1 provides an overview of the study’s design.

We chose the delayed-start design as it allows testing for intervention effects between and within both intervention arms [38,39] and helps to disentangle the effects of the intervention itself and the self-monitoring due to the daily diaries. Furthermore, including a baseline in both groups helps participants to get used to the daily diary approach and allows for longitudinal comparisons (before vs after reading the intervention materials).

**Table 1:** Overview of the intervention flow for the early and delayed intervention group. Links to view intervention materials were sent out at 8 AM on day 8 (early intervention group) or day 15 (delayed intervention group), and the links were valid for 12 hours.

Group	Day 0	Days 1-7	Days 8-14	Days 15-21	Day 22
Early intervention group	Entry questionnaire	Baseline measurement	Postintervention measurement	Postintervention measurement	Follow-up questionnaire
Delayed intervention group	Entry questionnaire	Baseline measurement	Baseline measurement	Postintervention measurement	Follow-up questionnaire

### Ethics Approval

Data collection for this study was performed between September 13, 2019, and October 10, 2019. The study was approved by the faculty’s ethics commission (ID MSHF0047). It was registered as a randomized trial in the German Clinical Trials Register (trial number DRKS00017379) and was preregistered in the Open Science Framework (OSF [41]).

### Sample Size Calculation

Sample size was determined before data collection using G*Power (version 3; Heinrich-Heine-Universität Düsseldorf) [42] based on an expected effect size of *f*=0.15 (with α=.05 and 1–β=.90). The calculation resulted in a required total sample size of 96 participants. Because we also planned to run multilevel models, a total sample size of 120 was targeted to increase the probability of achieving model convergence. As described further in the Results section, main analyses were performed using data from 149 participants.

### Recruitment, Eligibility Criteria, and Compensation

Participants were recruited via the institutes’ participant pool and social media postings. Eligibility criteria were a minimum age of 18 years, owning a smartphone with touch display and mobile internet access, and being able to answer daily questionnaires for 21 days. All participants received financial compensation for their participation: €3 (US $3.26) each for completing the entry and follow-up questionnaire, €4 (US $4.35) for responding to the intervention materials, €0.25 (US $0.27) for each completed daily questionnaire, and a bonus of €10 (US $10.87) for responding to more than 17 (80%) of the daily questionnaires (in total, up to €25.25; US $27.46).

### Measures

Table 2 provides an overview of the measures included in the different questionnaires of the intervention and informs about the internal consistency of the included scales. Internal consistency of all scales ranged between α=.74 and α=.88 and can be considered good.

**Table 2:** Overview of variables and Cronbach α of the scales measured in different intervention parts. Check marks indicate that a measure was used in that part of the study.

Measure	Entry	Intervention	Follow-up
Implicit Theories of Health Scale	✓ (α=.88)	✓ (α=.85)	✓ (α=.87)
Internal health locus of control	✓ (α=.76)		✓ (α=.83)
Chance health locus of control	✓ (α=.82)		✓ (α=.88)
Powerful others locus of control	✓ (α=.74)		✓ (α=.80)
Health-related self-efficacy	✓ (α=.85)		✓ (α=.83)
Health-related outcome expectancy	✓ (α=.77)		✓(α=.79)
Health status	✓		✓
Health value	✓		✓
Change motivation (self)	✓		✓
Change motivation (others)	✓		✓
Age	✓		
Gender	✓		
Height	✓		
Weight	✓		
Education	✓		
Occupation	✓		

### Implicit Theories

The Implicit Theories of Health Scale (ITHS) [7] was used to measure implicit theories of health. The scale consists of 6 items (eg, “You can substantially change your own health”). Three items represent an incremental theory of health, and 3 items represent an entity theory of health (which were recoded). Answers were given on 7-point Likert scales (1=strongly disagree to 7=strongly agree). A mean across all items was computed, with higher values indicating a stronger incremental theory.

#### Health-Promoting Behaviors

In the daily diaries, participants were asked every day whether they performed 10 health-promoting behaviors throughout the respective day (see Textbox 1; 0=no, 1=yes). We measured only behaviors (1) that could be performed during a regular day, (2) that were based on national recommendations from public health authorities (eg, Federal Centre for Health Education), and (3) that showed no ceiling or floor effect regarding the frequency of performing these behaviors, determined in a pretest (n=325). Concerning the latter, we did not include behaviors such as brushing one’s teeth or washing one’s hands because the pretest showed that almost all participants conducted these behaviors daily. The sum of performed health-promoting behaviors per day served as the primary outcome measure.

**Items to measure the frequency of performing health-promoting behaviors.**

**Nutrition**

- I ate at least 2 servings of fruit
- I ate at least 3 servings of vegetables
- I did not eat sweets
- I drank at least 2 liters of water

**Physical activity**

- I have been physically active for at least 30 minutes, so I started to sweat and/or was slightly out of breath
- I walked or cycled at least 6.5 kilometers
- I exercised

**Relaxation**

- I took some time to relax
- I slept for at least 7 hours

**Hygiene**

- I used dental floss

### Control Variables

Additionally, we measured health-related locus of control, self-efficacy, outcome expectancy, change motivation, health status, and health value. We included these variables to ensure (1) that the 2 intervention groups did not differ significantly regarding these constructs at baseline and (2) that the intervention only leads to changes in implicit theories and not the other constructs.

#### Health Locus of Control

The Health- and Illness-Related Locus of Control Questionnaire (Kontrollüberzeugung zu Krankheit und Gesundheit; KKG) [43] was used to measure health locus of control. The KKG consists of 21 items, all answered on 6-point Likert scales (1=strongly disagree to 6=strongly agree). Similar to its English equivalent [44], the KKG consists of 3 subscales (with 7 items each) to measure internal (eg, “If I do not feel well physically, I have to blame myself”), powerful others (eg, “If I feel well physically, then I owe it mainly to the advice and help of others”), and chance health locus of control (eg, “Whether my symptoms last longer depends mainly on chance”).

#### Health-Related Self-Efficacy

The Perceived Health Competence Scale [45] was used to measure health-related self-efficacy. The scale consists of 8 items (eg, “I'm generally able to accomplish my goals with respect to my health”) measured on 5-point Likert scales (1=strongly disagree to 5=strongly agree).

#### Health-Related Outcome Expectancy

Health-related outcome expectancy was measured using 6 statements to assess how much participants agree that specific health behaviors can influence one’s own health (“Your health is strongly influenced by...eating behavior,... physical activity and exercising,... consumption of harmful substances,... enough sleep and relaxation,… personal and dental hygiene,... regular doctor visits, and checkups”). Participants’ agreement was assessed via 7-point Likert scales (1=strongly disagree to 7=strongly agree).

#### Further Health-Related Variables

Single items measured current subjective health status (“How would you describe your health status in general?”; 1=bad to 7=excellent), health value (“How important is your health to you?”; 1=not at all important to 7=very important), and the extent to which participants think that they should change their health from their point of view (“It is important to me to change something about my health”; 1=strongly disagree to 7=strongly agree) and from the perspective of others (“From the perspective of others, I should change something about my health”; 1=strongly disagree to 7=strongly agree).

### Intervention Materials

Participants received a link to the intervention materials on their smartphones either after 7 (early intervention group) or 14 days (delayed intervention group) of baseline measurement. The links to view the intervention materials were sent out at 8 AM on the specified day (day 8 or 15), and the individual links were available until 8 PM on that day. Like other interventions to promote incremental theories [12,20,22], the intervention materials consisted of informative, exemplary, and reflective components. More precisely, the intervention materials included (1) a (fictitious) newspaper article that described health as mainly influenced by lifestyle and engagement in health-promoting behavior [7], (2) three fictitious blog posts in which individuals reported positive health changes, (3) an essay priming in which participants were asked to describe health changes in their lives, and (4) an article that focused on the benefits of beliefs in changeability in other domains. The materials are available in the OSF repository [41]. After reading the articles and the 3 blog posts, participants answered one question regarding the content of the materials as an attention check. In addition, an independent rater checked the content of the essays to determine whether the participants followed the task description. On the basis of this, we calculated an attention check score, ranging from 1 to 4, with 4 points indicating that all content questions were answered correctly and that the essay fitted the instruction.

## Results

### Participants

**Figure 1:**
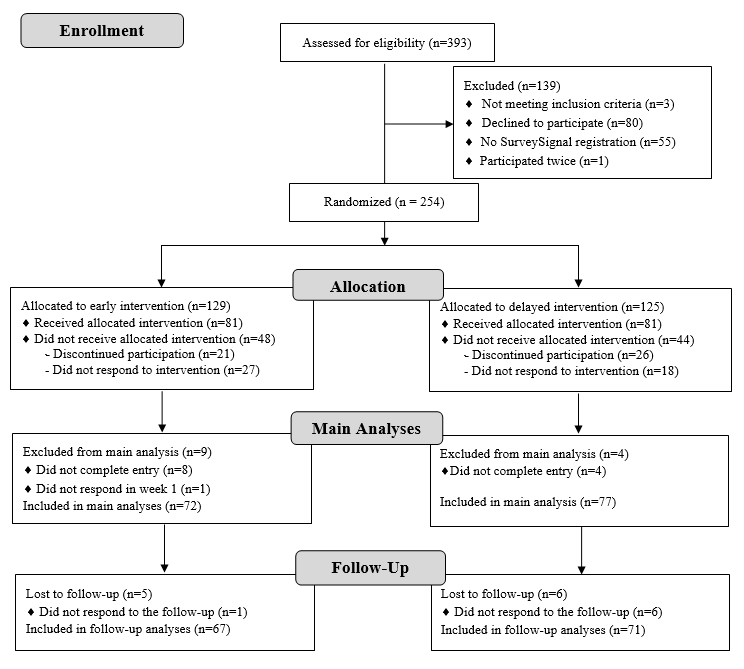
CONSORT (Consolidated Standards of Reporting Trials) flow diagram.

Initially, 393 participants were screened regarding eligibility criteria (see the CONSORT [Consolidated Standards of Reporting Trials] flow diagram. flow diagram in Figure 1). A total of 254 participants were randomly assigned to 1 of the 2 intervention arms (early vs delayed intervention). As some participants discontinued their participation or did not respond to the intervention materials, 162 participants received the allocated intervention (81 participants in both intervention arms). Participants were excluded from data analysis when they did not complete the entry questionnaire (early intervention: n=8; delayed intervention: n=4) or did not respond to the daily diaries during the first week (early intervention: n=1). No participants were excluded based on their attention check scores, as all remaining participants scored 2 points or higher. Further, a regression analysis revealed that the attention check scores did not have an impact on the change in implicit theories measured in the entry questionnaire versus directly after seeing the intervention materials (*b*=0.19; *t*_147_=0.73, SE 0.26; *P*=.47; 95% CI −0.32 to 0.70). Consequently, main analyses were performed with 149 participants (early intervention: n=72; delayed intervention: n=77). The mean age of the analyzed sample was 30.58 (SD 9.71) years, with 52% (79/149) female and 47% (70/149) male participants. Table 3 includes other demographic characteristics, and the CONSORT flow diagram (Figure 1) provides an overview of participant flow and informs about dropout reasons in each intervention group. For additional follow-up analyses, data of 138 participants were available.

**Table 3:** Baseline characteristics of participants in total and by intervention group (n=149).

Characteristics		Overall	Early (n=72)	Delayed (n=77)	Condition difference		*P* value	
Age (years), mean (SD)		30.58 (9.71)	31.31 (10.44)	29.91 (8.98)	0.88 (147)^a^		.38	
Implicit theories, mean (SD)		5.29 (0.99)	5.36 (1.06)	5.22 (0.92)	0.86 (147)^a^		.39	
Internal locus, mean (SD)		3.78 (0.70)	3.87 (0.72)	3.69 (0.67)	1.61 (147)^a^		.11	
Powerful others locus, mean (SD)		2.90 (0.73)	2.92 (0.73)	2.88 (0.73)	0.34 (147)^a^		.73	
Chance locus, mean (SD)		2.35 (0.74)	2.39 (0.79)	2.32 (0.70)	0.60 (147)^a^		.55	
Self-efficacy, mean (SD)		3.52 (0.68)	3.54 (0.71)	3.51 (0.64)	0.26 (147)^a^		.80	
Outcome-expectancy, mean (SD)		5.56 (0.83)	5.63 (0.81)	5.49 (0.84)	1.02 (147)^a^		.31	
Height (in meters), mean (SD)		174.60 (9.63)	175.21 (9.38)	174.04 (9.89)	0.74 (147)^a^		.46	
Weight (in kilogram), mean (SD)		77.82 (18.88)	78.18 (21.26)	77.49 (16.51)	0.22 (146)^a^		.82	
BMI, mean (SD)		25.37 (5.17)	25.22 (5.43)	25.52 (4.95)	0.35 (146)^a^		.72	
Health status, mean (SD)		4.99 (1.15)	4.97 (1.13)	5.00 (1.18)	0.15 (147)^a^		.88	
Health value, mean (SD)		6.11 (0.98)	5.97 (1.07)	6.23 (0.87)	1.64 (147)^a^		.10	
Change motivation (self), mean (SD)		5.50 (1.22)	5.43 (1.27)	5.56 (1.18)	0.64 (147)^a^		.52	
Change motivation (others), mean (SD)		3.37 (1.77)	3.44 (1.68)	3.30 (1.86)	0.50 (147)^a^		.62	
**Gender, n (%)**						2.51 (1)^b^		.11
	Male	70 (47)	29 (40)	41 (53)				
	Female	79 (53)	43 (60)	36 (47)				
**Education, n (%)**						3.66 (4)^b^		.45
	Lower secondary school	4 (2.7)	2 (3)	2 (3)				
	Secondary school	10 (6.7)	4 (6)	6 (8)				
	Entitlement to study at a university of applied sciences	3 (2)	3 (4)	0 (0)				
	Higher education entrance qualification (“Abitur”)	61 (40.9)	28 (39)	33 (43)				
	University degree	71 (47.7)	35 (49)	36 (47)				
**Occupation, n (%)**						8.90 (6)^b^		.18
	Full-time employed	40 (26.8)	24 (33)	16 (21)				
	Part-time employed	13 (8.7)	4 (6)	9 (12)				
	Studying	81 (54.4)	36 (50)	45 (58)				
	Stay-at-home spouse	3 (2)	0 (0)	3 (4)				
	Retired	5 (3.4)	3 (4)	2 (3)				
	Occupational disability	3 (2)	2 (3)	1 (1)				
	Other	4 (2.7)	3 (4)	1 (1)				

^a^These values are the *t* (*df*).

^b^These values are the chi-square.

### Precursory Analyses

As depicted in Table 2, there were no significant differences between the 2 intervention groups regarding demographics or other measures included in the entry questionnaire, suggesting that the randomization was successful. Participants answered a total of 3015 daily questionnaires; on average, each participant answered 20.23 (SD 1.42, range 12-21) questionnaires.

As a manipulation check, a paired-samples 2-tailed *t* test with ITHS scores measured in the entry questionnaire and ITHS scores after responding to the intervention materials was performed to test whether the intervention led participants to adopt a stronger incremental theory. This *t* test revealed that participants reported a stronger incremental theory after responding to intervention materials (mean 5.58, SE 0.07), compared with the entry questionnaire (mean 5.29, SE 0.08; *t*_148_=4.07, SE 0.07, *P*<.001, 95% CI 0.15-0.43, *d*=0.33). Further, a 2 (intervention group: early vs delayed) by 2 (time of assessment: entry questionnaire vs directly after seeing the intervention materials) mixed ANOVA revealed that the intervention led to an increase in incremental theories in both groups indicated by a significant main effect of time of implicit theories assessment (*F*_1,147_=16.42; *P*<.001; η_p_^2^=.10), a nonsignificant main effect of intervention group (*F*_1,147_=1.19; *P*=.28; η_p_^2^=.01), and a nonsignificant interaction (*F*_1,147_=0.02; *P*=.89; η_p_^2^<.01).

### Main Analyses

**Figure 2:**
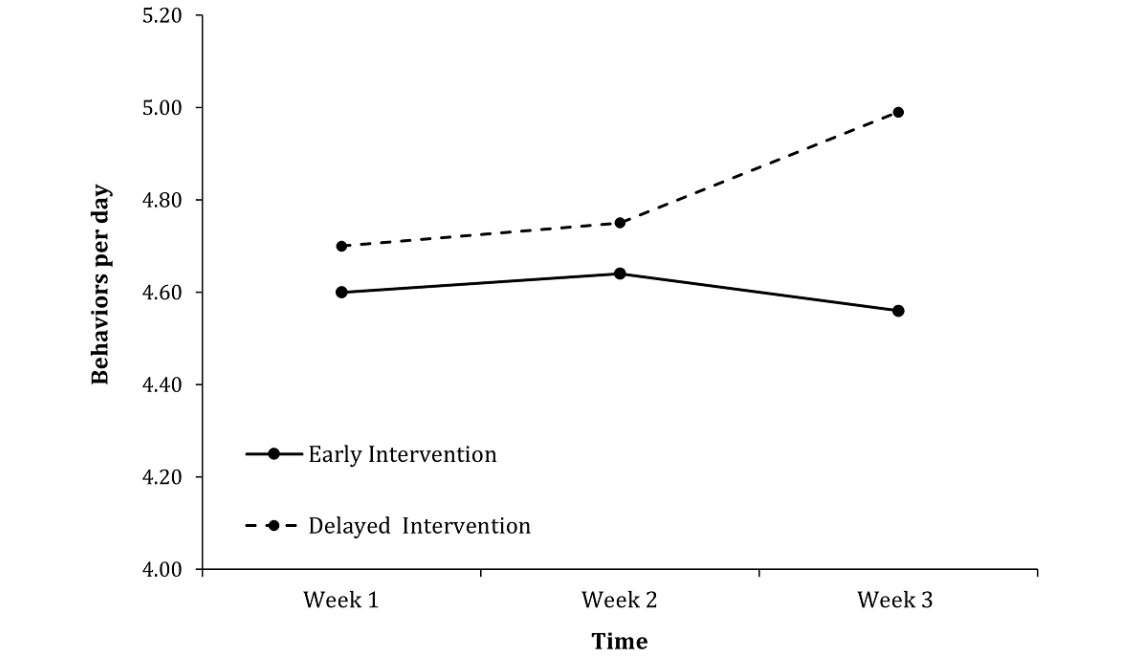
Mean number of performed health-promoting behaviors per day aggregated on a weekly level for both intervention groups.

Based on the preregistration [41], a mixed ANOVA was conducted to test whether the intervention increased the frequency of performing health-promoting behaviors on a weekly level. As within-subject factor, the mean number of health-promoting behaviors per day was aggregated for every week, and intervention group (early versus delayed) was entered as between-subject factor. The results of the mixed ANOVA showed no significant main effect of the intervention group (*F*_1,147_=0.92; *P*=.34; η_p_^2^=.01). There was also no significant difference between mean daily performed behaviors per week (*F*_2,294_=1.46; *P*=.23; η_p_^2^=.01). However, a significant interaction between the intervention group and week emerged (*F*_2,294_=3.06; *P*=.048; η_p_^2^=.02). Table 4 shows marginal means, SEs, and 95% CIs for daily performed behaviors per week for both intervention groups, and Figure 2 illustrates the interaction.

**Table 4:** Marginal means, SEs, and 95% CIs for mean performed health behaviors per day as a result of the condition × time in a mixed ANOVA.

Condition		Mean (SE)	95% CI
**Early intervention**			
	Week 1	4.60 (0.17)	4.27-4.92
	Week 2	4.64 (0.18)	4.29-4.99
	Week 3	4.56 (0.18)	4.21-4.91
**Delayed intervention**			
	Week 1	4.70 (0.16)	4.38-5.01
	Week 2	4.75 (0.17)	4.42-5.09
	Week 3	4.99 (0.17)	4.65-5.33

As the marginal means and their corresponding confidence intervals did not show significant differences between both groups in any week, the preregistered *t* tests were not conducted. However, as depicted in Figure 2 and indicated by the significant interaction between time and condition, the delayed intervention group may have benefitted from viewing the intervention materials. Therefore, the preregistered multilevel models were performed to test whether the intervention increased the frequency of performing health-promoting behaviors on a daily level. Day was treated as the level 1 unit and participant as the level 2 unit. Intervention status (0=before intervention, 1=after intervention) served as the level 1 predictor, whereas the number of performed health-promoting behaviors served as the level 1–dependent variable. A deviance test was conducted for each analysis to test whether a random-slope or a random-intercept model results in a better model fit. Across both intervention groups, the better fitting random-slope model showed an increase in the number of performed health-promoting behaviors after responding to the intervention materials (*b*=0.14; *t*_146.65_=2.06, SE 0.07; *P*=.04, 95% CI 0.01-0.28). Because of the significant interaction between time and condition found in the mixed ANOVA, additional multilevel models were conducted separately for both intervention groups. These multilevel models revealed that the effect of the intervention only appeared for the delayed intervention group (random-intercept model, *b*=0.27; *t*_1492.37_=3.50, SE 0.08; *P*<.001; 95% CI 0.12-0.42). In contrast, no difference before and after the intervention was detected for the early intervention group (random-slope model, *b*=0.02; *t*_69.23_=0.14, SE 0.11; *P*=.89; 95% CI −0.2 to 0.23). The information criteria and results of the likelihood-ratio tests for comparing the multilevel models are available in Table S1 in Multimedia Appendix 1.

### Additional Analyses

Additional analyses revealed that participants did also report a stronger incremental theory in the follow-up questionnaire (mean 5.42, SE 0.08) compared with the entry questionnaire (mean 5.24, SE 0.08; *t*_137_=2.42, SE 0.07; *P*=.02; 95% CI 0.03-0.32; *d*=0.20). Furthermore, participants reported a stronger internal health locus of control in the follow-up questionnaire (mean 3.92, SE 0.06) compared with the entry questionnaire (mean 3.78, SE 0.06; *t*_137_=3.17, SE 0.04; *P*=.002; 95% CI 0.05-0.23; *d*=0.28). To test whether our intervention also led to an increase in health-promoting behaviors when controlling for the change in internal health locus of control, we performed additional multilevel models, including the change in internal health locus of control as the level 2 predictor. Table 5 shows that these robustness checks led to the same conclusions as the multilevel models described in the previous section. For all other health-related variables, no significant difference between the entry and follow-up questionnaire emerged.

**Table 5:** Multilevel models including the change in internal health locus of control as an additional level 2 predictor to determine whether our intervention effects are robust when controlling for the internal health locus of control change.

Variable		*B*	SE	*t* (*df*)	*P* value	95% CI
**Predicting health-promoting behaviors per day (across conditions)**						
	Intercept	4.70	0.11	39.54 (135.72)	<.001	4.46 to 4.93
	Time (0=before intervention; 1=after intervention)	0.15	0.07	2.15 (135.78)	.03	0.01 to 0.29
	Change (internal health locus)	0.13	0.22	0.61 (136.14)	.54	−0.29 to 0.56
**Predicting health-promoting behaviors per day (early intervention group**)						
	Intercept	4.69	0.18	26.07 (63.58)	<.001	4.33 to 5.05
	Time (0=before intervention; 1=after intervention)	0.04	0.11	0.37 (64.64)	.71	−0.18 to 0.26
	Change (internal health locus)	0.40	0.33	1.22 (65.83)	.23	−0.25 to 1.05
**Predicting health-promoting behaviors per day (delayed intervention group)**						
	Intercept	4.78	0.17	27.54 (72.11)	<.001	4.44 to 5.13
	Time (0=before intervention, 1=after intervention)	0.26	0.08	3.30 (1374.34)	<.001	0.11 to 0.42
	Change (internal health locus)	−0.14	0.30	−0.46 (68.83)	.65	−0.74 to 0.46

## Discussion

### Principal Findings

The present research aimed to examine whether a smartphone-based intervention to foster incremental theories of health increases the frequency of performing health-promoting behaviors in daily life measured via ecological momentary assessment. Indicated by our manipulation check, we found that the intervention led to stronger incremental theories of health. Furthermore, across conditions, participants showed a significant increase in the frequency of performing health-promoting behaviors after being confronted with the intervention materials. However, this effect was only driven by the delayed intervention group, whereas the early intervention group did not increase in health-promoting behaviors.

One possible explanation for why the effectiveness of the intervention differed between intervention groups may be that incremental theories may only have a beneficial effect in the long run. As depicted in Figure 2, both intervention groups showed a slight increase in health-promoting behaviors between the first and second week. This could be due to the involvement of self-monitoring evoked by the daily diaries, which can have an intervention effect itself [46]. Being confronted with the intervention materials at an early stage (week 1) did not seem to have any additional motivational benefit for the early intervention group. On the other hand, the delayed intervention group was confronted with the fact that they *only* showed half of the measured behaviors every day for 2 weeks. Being shown the intervention materials at this point in time (week 2) had an additional motivating effect beyond the effect of self-monitoring. Instead, the early intervention group shows a decline in health-promoting behaviors in the third week without an additional boost in motivation. This pattern fits Yeager and Dweck’s [47] argument that incremental theories are especially helpful when challenges arise (like continually maintaining the motivation to engage in health-promoting behaviors over 3 weeks). As this explanation is only speculative, further research is needed to investigate whether the observed time-dependent effectiveness of the intervention replicates consistently or has resulted by chance.

Although no intervention effect emerged for the early intervention group, the introduced intervention led to changes in health behavior for the delayed intervention group. Thus, this study is the first to show that implicit theories of health can be influenced through an intervention delivered in people’s daily lives. It provides further evidence of the relevance of these theories for health behavior change across multiple health domains and extends the existing correlative and experimental findings on implicit theories of health [7-10]. The results show that even a one-shot implicit theory intervention via web-based materials can increase engagement in health-promoting behaviors. Hence, this approach represents a time-, effort-, and cost-efficient way for health promotion. This study also increases the ecological validity of previous findings by measuring health-promoting behaviors using ecological momentary assessment [37]. We show that incremental theories are relevant not only in laboratory research or one-shot web-based questionnaires but also to everyday behavior.

Additional analyses revealed that the intervention-based increase in incremental theories of health is not just short-term, as participants also reported stronger incremental theories in the follow-up questionnaire (compared with the entry questionnaire). In addition, the present intervention led to a stronger internal health locus of control. This is consistent with previous findings showing that a stronger internal health locus of control mediates the effect of an implicit theories of health manipulation on health-promoting outcomes [7]. It also fits findings that an incremental theory of personality intervention increases primary control in the context of mental illness [20,21]. It remains to be tested whether the change in internal health locus of control stems from the intervention materials, the daily diary assessment, or the combination of both.

### Limitations and Generalizability

We chose a delayed-start design to test for intervention effects between and within both intervention groups. However, we did not find a difference in health behavior engagement between the 2 groups in the second week of our assessment. Future trials should include a no-treatment control group that does not receive intervention materials. This approach makes it possible to determine whether the pre-post difference in behavior in the delayed intervention group appeared because of the intervention materials or because of the combination of the intervention materials with daily diaries.

We incorporated ecological momentary assessment in which participants were asked daily whether they performed 10 health-promoting behaviors using a simple yes-no format. This format allows for more objective and reliable measures of self-reported health behavior engagement with less recall and retrieval bias compared with standard forms of measurement in which individuals usually have to recall behavior engagement over longer periods (like weeks or months) [37]. However, these self-reports can still be affected by social desirability. Therefore, future studies could incorporate more objective measurements of health behavior engagement, like taking pictures of meals to measure eating behavior or using smartwatches or other devices to measure physical activity (for a physical activity example, see Henderson et al [48]).

Regarding the generalizability of findings, it is essential to note that the surveyed sample differs from the general population, especially regarding age, educational level, and student proportion. Participation in the study required owning a smartphone with internet access, and recruitment was realized via social media and mailing lists. This has limited the studies’ accessibility for individuals of older age. Moreover, participants reported high values on other health-relevant measures at baseline (eg, health status, health change motivation, and self-efficacy; see Table 1). It may have been easier for individuals with such characteristics to engage in health-promoting behaviors or adopt new behavioral routines. On the other hand, the present intervention even led to positive changes in health-promoting behaviors for individuals already starting with such advantageous conditions. Thus, individuals lacking these attributes might benefit even more from the intervention introduced.

Recently, the relevance of implicit theories interventions has been seriously tackled in 2 meta-analyses concluding that they only produce weak effects in educational settings [49]. According to classic convictions [50], the reported effect sizes or regression coefficients in the present research also fall in this category. However, it has been argued that these convictions should be used with caution, and effect sizes should be evaluated considering the area or context investigated [50,51]. Especially in health or educational research, even small effects can have far-reaching consequences when evaluated in a broader context [23,51].

This study is in line with the majority of research showing that a stronger incremental theory leads to beneficial outcomes [5]. However, holding an entity theory can be instrumental under specific circumstances. A stronger incremental theory of health not only implies that one’s health can improve but also means that one’s health might worsen. For this reason, an incremental theory would be less adaptive when a prevention focus is present [52,53], that is, when one is trying to conserve a given health status. For individuals being confronted with the process of aging or suffering from long-lasting diseases, it may be more beneficial to believe in the stability of health.

### Theoretical and Practical Implications

As introduced in the present research, addressing implicit theories of health serves as a new approach for achieving positive health behavior change. The expanding research of implicit theories in the health domain [7-10] may guide the development of health interventions or could be integrated into health education. Nevertheless, further steps are needed to test whether the present findings replicate and can be generalized. First, direct replications could test whether the effectiveness of an incremental theories intervention is indeed time-sensitive, as demonstrated in this study. In addition, future studies should also focus on testing the longevity of the intervention effect. Investigating whether the increase in incremental theories can be sustained over longer periods and whether this increase translates into sustained improvements in health behaviors is essential. Therefore, studies with increased follow-up periods are necessary to test the longevity of the intervention effect and the respective impact on health behaviors. Next, conceptual replications could investigate what modes of delivering an implicit theories intervention are most effective and for whom. For example, the effectiveness might be higher when the changeability of health is emphasized several times over an extended course of time. A useful tool in this respect could be an app that tracks and visualizes changes in one’s health behavior over time. Finally, the generalizability to different populations and different health behavior measures needs to be ensured.

Research on implicit theories of health would also benefit greatly from examining the antecedents and determinants that lead to adopting an incremental versus entity theory [7]. Auster-Gussman and Rothman [24] found that incremental theories of body weight are more common among young and White individuals with a higher level of income and education. These variables, as well as one’s medical history or that of close others, should also play a significant role in the formation of implicit theories [7]. This study shows that even a rather young and educated sample with high self-reported health and high incremental theories at baseline benefits from an incremental theories intervention. Effects might be stronger when studying population groups with higher entity theories of health.

### Conclusions

This study is the first randomized trial demonstrating that incremental theories of health can increase because of a single-session smartphone-based intervention. Contrary to our assumptions, the intervention only led to an increase in performing health-promoting behaviors when delivered at a later point in time. Further studies are crucial to assure whether the observed time-dependent variation in effectiveness replicates. Incremental theories interventions might be most effective for individuals holding a stronger entity theory of health. Factors that favor the development of an entity theory of health should be investigated to identify population groups that would benefit most from the interventional approach introduced in this paper.

## Appendix

Multimedia Appendix 1 Table S1. Regression coefficients and information criteria for comparing the multilevel models to predict health behavior engagement per day.

Multimedia Appendix 2 CONSORT-eHEALTH checklist (V 1.6.1).

## Abbreviations

BMI: body mass index
CONSORT: Consolidated Standards of Reporting TrialsITHS: Implicit Theories of Health Scale
KKG: Kontrollüberzeugung zu Krankheit und Gesundheit (Health- and Illness- Related Locus of Control Questionnaire)
OSF: Open Science Framework

## References

[1] (2020). Noncommunicable disease: Unhealthy diet. World Health Organization.

[2] van Dam RM, Li T, Spiegelman D, Franco OH, Hu FB (2008). Combined impact of lifestyle factors on mortality: prospective cohort study in US women. BMJ.

[3] Leventhal H, Cameron LD (2008). The Self-Regulation of Health and Illness Behaviour.

[4] Hofmann W, Friese M, Wiers RW (2008). Impulsive versus reflective influences on health behavior: a theoretical framework and empirical review. Health Psychol Rev.

[5] Burnette JL, O'Boyle EH, VanEpps EM, Pollack JM, Finkel EJ (2013). Mind-sets matter: a meta-analytic review of implicit theories and self-regulation. Psychol Bull.

[6] Dweck CS (2000). Self-theories: Their Role in Motivation, Personality, and Development.

[7] Schreiber M, Job V, Dohle S (2020). Is your health malleable or fixed? The influence of implicit theories on health-related attitudes and behaviour. Psychol Health.

[8] Thomas FN, Burnette JL, Hoyt CL (2019). Mindsets of health and healthy eating intentions. J Appl Soc Psychol.

[9] Bunda K, Busseri MA (2019). Lay theories of health, self-rated health, and health behavior intentions. J Health Psychol.

[10] Zhang N, Kou Y (2022). Implicit theories of health, consideration of future consequences, and engagement in health protective behaviors during the COVID-19 pandemic in China. J Health Psychol.

[11] Hong Y-Y, Chiu C-Y, Dweck CS, Kernis MH (1995). Implicit theories of intelligence: Reconsidering the role of confidence in achievement motivation. Efficacy, Agency, and Self-esteem.

[12] Blackwell LS, Trzesniewski KH, Dweck CS (2007). Implicit theories of intelligence predict achievement across an adolescent transition: a longitudinal study and an intervention. Child Dev.

[13] Dweck CS, Leggett EL (1988). A social cognitive approach to motivation and personality. Psychol Rev.

[14] Job V, Dweck CS, Walton GM (2010). Ego depletion--is it all in your head? implicit theories about willpower affect self-regulation. Psychol Sci.

[15] Job V, Walton GM, Bernecker K, Dweck CS (2015). Implicit theories about willpower predict self-regulation and grades in everyday life. J Pers Soc Psychol.

[16] Chiu C-Y, Dweck CS, Tong JY, Fu JH (1997). Implicit theories and conceptions of morality. J Pers Soc Psychol.

[17] Levy SR, Stroessner SJ, Dweck CS (1998). Stereotype formation and endorsement: The role of implicit theories. J Pers Soc Psychol.

[18] Knee CR (1998). Implicit theories of relationships: assessment and prediction of romantic relationship initiation, coping, and longevity. J Pers Soc Psychol.

[19] Dweck CS, Kruglanski AW, Higgins ET, van Lange PAM (2012). Implicit theories. Theories of Social Psychology.

[20] Schleider JL, Weisz JR (2016). Reducing risk for anxiety and depression in adolescents: effects of a single-session intervention teaching that personality can change. Behav Res Ther.

[21] Schleider J, Weisz J (2018). A single-session growth mindset intervention for adolescent anxiety and depression: 9-month outcomes of a randomized trial. J Child Psychol Psychiatry.

[22] Burnette JL, Finkel EJ (2012). Buffering against weight gain following dieting setbacks: an implicit theory intervention. J Exp Soc Psychol.

[23] Yeager DS, Hanselman P, Walton GM, Murray JS, Crosnoe R, Muller C, Tipton E, Schneider B, Hulleman CS, Hinojosa CP, Paunesku D, Romero C, Flint K, Roberts A, Trott J, Iachan R, Buontempo J, Yang SM, Carvalho CM, Hahn PR, Gopalan M, Mhatre P, Ferguson R, Duckworth AL, Dweck CS (2019). A national experiment reveals where a growth mindset improves achievement. Nature.

[24] Auster-Gussman LA, Rothman AJ (2018). Understanding the prevalence and correlates of implicit theories of weight in the United States: Insights from a nationally representative sample. Psychol Health.

[25] Burnette JL (2010). Implicit theories of body weight: entity beliefs can weigh you down. Pers Soc Psychol Bull.

[26] Taber JM, Klein WMP, Persky S, Ferrer RA, Kaufman AR, Thai CL, Harris PR (2017). Interest in and reactions to genetic risk information: the role of implicit theories and self-affirmation. Soc Sci Med.

[27] Lyons C, Kaufman AR, Rima B (2015). Implicit theories of the body among college women: implications for physical activity. J Health Psychol.

[28] Orvidas K, Burnette JL, Russell VM (2018). Mindsets applied to fitness: growth beliefs predict exercise efficacy, value and frequency. Psychol Sport Exerc.

[29] Fitz CC, Kaufman A, Moore PJ (2015). Lay theories of smoking and young adult nonsmokers' and smokers' smoking expectations. J Health Psychol.

[30] Thai CL, Coa KI, Kaufman AR (2018). Implicit theories of smoking and association with current smoking status. J Health Psychol.

[31] Sridharan V, Shoda Y, Heffner JL, Bricker J (2019). Addiction mindsets and psychological processes of quitting smoking. Subst Use Misuse.

[32] Burnette JL, Forsyth RB, Desmarais SL, Hoyt CL (2019). Mindsets of addiction: implications for treatment intentions. J Soc Clin Psychol.

[33] Schleider JL, Weisz JR (2016). Implicit theories relate to youth psychopathology, but how? A longitudinal test of two predictive models. Child Psychiatry Hum Dev.

[34] Plaks JE, Levy SR, Dweck CS (2009). Lay theories of personality: cornerstones of meaning in social cognition. Soc Pers Psychol Compass.

[35] Heron KE, Smyth JM (2010). Ecological momentary interventions: incorporating mobile technology into psychosocial and health behaviour treatments. Br J Health Psychol.

[36] Stark AL, Geukes C, Dockweiler C (2022). Digital health promotion and prevention in settings: scoping review. J Med Internet Res.

[37] Shiffman S, Stone AA (1998). Introduction to the special section: ecological momentary assessment in health psychology. Health Psychol.

[38] D'Agostino RB (2009). The delayed-start study design. N Engl J Med.

[39] Parkinson Study Group (2004). A controlled, randomized, delayed-start study of rasagiline in early parkinson disease. Arch Neurol.

[40] Hofmann W, Patel PV (2014). SurveySignal: a convenient solution for experience sampling research using participants' own smartphones. Soc Sci Comput Rev.

[41] Schreiber M, Dohle S (2019). Implicit Theories Intervention for Multiple Health Behavior Change.

[42] Faul F, Erdfelder E, Lang A-G, Buchner A (2007). G*Power 3: a flexible statistical power analysis program for the social, behavioral, and biomedical sciences. Behav Res Methods.

[43] Lohaus A, Schmitt GM (1989). Kontrollüberzeugungen zu krankheit und gesundheit (KKG): bericht über die entwicklung eines testverfahrens control beliefs about illness and health (KKG): report on the development of a measure. Diagnostica.

[44] Wallston KA, Wallston BS, DeVellis R (1978). Development of the Multidimensional Health Locus of Control (MHLC) scales. Health Educ Monogr.

[45] Smith MS, Wallston KA, Smith CA (1995). The development and validation of the Perceived Health Competence Scale. Health Educ Res.

[46] Michie S, Richardson M, Johnston M, Abraham C, Francis J, Hardeman W, Eccles MP, Cane J, Wood CE (2013). The behavior change technique taxonomy (V1) of 93 hierarchically clustered techniques: building an international consensus for the reporting of behavior change interventions. Ann Behav Med.

[47] Yeager DS, Dweck CS (2020). What can be learned from growth mindset controversies?. Am Psychol.

[48] John-Henderson NA, Tintzman CS, Counts CJ, Mueller CM (2021). Health mindsets as a predictor of physical activity and body mass index in american indian college students. J Health Psychol.

[49] Sisk VF, Burgoyne AP, Sun J, Butler JL, Macnamara BN (2018). To what extent and under which circumstances are growth mind-sets important to academic achievement? Two meta-analyses. Psychol Sci.

[50] Cohen J (1988). Statistical Power Analysis for the Behavioral Sciences.

[51] Funder DC, Ozer DJ (2019). Evaluating effect size in psychological research: sense and nonsense. Adv Meth Pract Psychol Sci.

[52] Sue-Chan C, Wood RE, Latham GP (2010). Effect of a coach's regulatory focus and an individual's implicit person theory on individual performance. J Manag.

[53] Sevincer AT, Kluge L, Oettingen G (2013). Implicit theories and motivational focus: desired future versus present reality. Motiv Emot.

