# Supplementary material for: A Smartphone-Based Implicit Theories Intervention for Health Behavior Change: Randomized Trial
Source: JMIR Mhealth Uhealth. 2023 Jun 15;11:e36578. doi: 10.2196/36578 (PMC10337348; doi:10.2196/36578)
Supplement: Multimedia Appendix 1 [file mhealth_v11i1e36578_app1.docx]

Table S1. Regression coefficients and information criteria for comparing the multilevel models to predict health behavior engagement per day.

|  | Null Model | | Random Intercept | Random Slope |
| --- | --- | --- | --- | --- |
| **Predicting health-promoting behaviors per day (across conditions)** | | | | |
| Intercept | 4.73; *P*<.001 | | 4.67; *P*<.001 | 4.68; *P*<.001 |
| *b* (0 = *pre-*; 1 = *post-intervention*) | — | | 0.14 *P*=.01 | 0.14; *P*=.04 |
| AIC | 10968.16 | | 10963.31 | 10935.95 |
| BIC | 10986.21 | | 10987.37 | 10972.05 |
| -2LL | 10962.16 | | 10955.31 | 10923.95 |
| *df* | 3 | | 4 | 6 |
| Comparison (NM vs RI) | — | | X²_1_=6.85; *P*=.009 | X²_3_=38.17; *P*<.001 |
| Comparison (RI vs RS) | — | | — | X²_2_=31.36; *P*<.001 |
| **Predicting health-promoting behaviors per day (delayed intervention)** | | | | |
| Intercept | | 4.82; *P*<.001 | 4.73; *P*<.001 | 4.73; *P*<.001 |
| *b* (0 = *pre-*; 1 = *post-intervention*) | | — | 0.27; *P*<.001 | 0.27; *P*=.003 |
| AIC | | 5809.88 | 5799.67 | 5798.45 |
| BIC | | 5825.96 | 5821.11 | 5830.61 |
| -2LL | | 5803.88 | 5791.67 | 5786.45 |
| *d*f | | 3 | 4 | 6 |
| Comparison (NM vs RI | | — | X²_1_=12.21; *P*<.001 | X²_3_=17.43; *P<*.001 |
| Comparison (RI vs RS) | | — | — | X²_2_=5.22; *P*=.08 |
| **Predicting health-promoting behaviors per day (early intervention)** | | | | |
| Intercept | | 4.65; *P*<.001 | 4.65; *P*<.001 | 4.64; *P*<.001 |
| *b* (0 = *pre-*; 1 = *post-intervention*) | | -- | 0.01; *P*=.93 | 0.02; *P*=.89 |
| AIC | | 5149.58 | 5151.57 | 5123.63 |
| BIC | | 5165.43 | 5172.71 | 5155.34 |
| -2LL | | 5143.58 | 5143.57 | 5111,63 |
| *df* | | 3 | 4 | 6 |
| Comparison (NM vs RI) | | — | X²_1_=0.01; *P*=.92 | X²_3_=31.95, *P*<.001 |
| Comparison (RI vs RS) | | — | — | X²_2_=31.94; *P*<.001 |

Note. AIC = Akaike Information Criterion; BIC = Baysian Information Criterion; LL = Log Likelihood; NM = Null Model; RI = Random Intercept Model; RS = Random Slope Model.
