# Supplementary material for: A Smartphone-Based Implicit Theories Intervention for Health Behavior Change: Randomized Trial
Source: JMIR Mhealth Uhealth. 2023 Jun 15;11:e36578. doi: 10.2196/36578 (PMC10337348; doi:10.2196/36578)
Supplement: Multimedia Appendix 2 [file mhealth_v11i1e36578_app2.pdf]

CONSORT-EHEALTH (V 1.6.1) - Submission/Publication Form

The CONSORT-EHEALTH checklist is intended for authors of randomized trials evaluating web-based and Internet-based applications/interventions, including mobile interventions, electronic games (incl multiplayer games), social media, certain telehealth applications, and other interactive and/or networked electronic applications. Some of the items (e.g. all subitems under item 5 - description of the intervention) may also be applicable for other study designs.

The goal of the CONSORT EHEALTH checklist and guideline is to be
a) a guide for reporting for authors of RCTs,
b) to form a basis for appraisal of an ehealth trial (in terms of validity)

CONSORT-EHEALTH items/subitems are MANDATORY reporting items for studies published in the Journal of Medical Internet Research and other journals / scientific societies endorsing the checklist.

Items numbered 1., 2., 3., 4a., 4b etc are original CONSORT or CONSORT-NPT (non-pharmacologic treatment) items.
Items with Roman numerals (i., ii, iii, iv etc.) are CONSORT-EHEALTH extensions/clarifications.

As the CONSORT-EHEALTH checklist is still considered in a formative stage, we would ask that you also RATE ON A SCALE OF 1-5 how important/useful you feel each item is FOR THE PURPOSE OF THE CHECKLIST and reporting guideline (optional).

Mandatory reporting items are marked with a red *.
In the textboxes, either copy & paste the relevant sections from your manuscript into this form - please include any quotes from your manuscript in QUOTATION MARKS,
or answer directly by providing additional information not in the manuscript, or elaborating on why the item was not relevant for this study.

YOUR ANSWERS WILL BE PUBLISHED AS A SUPPLEMENTARY FILE TO YOUR PUBLICATION IN JMIR AND ARE CONSIDERED PART OF YOUR PUBLICATION (IF ACCEPTED).
Please fill in these questions diligently. Information will not be copyedited, so please use proper spelling and grammar, use correct capitalization, and avoid abbreviations.

DO NOT FORGET TO SAVE AS PDF _AND_ CLICK THE SUBMIT BUTTON SO YOUR ANSWERS ARE IN OUR DATABASE !!!

Citation Suggestion (if you append the pdf as Appendix we suggest to cite this paper in the caption):
Eysenbach G, CONSORT-EHEALTH Group
CONSORT-EHEALTH: Improving and Standardizing Evaluation Reports of Web-based and Mobile Health Interventions
J Med Internet Res 2011;13(4):e126

URL: http://www.jmir.org/2011/4/e126/
doi: 10.2196/jmir.1923
PMID: 22209829

mike.schreiber.1992@gmail.com Konto wechseln

Nicht freigegeben

Entwurf gespeichert

* Gibt eine erforderliche Frage an

Your name *

First Last

Mike Schreiber

Primary Affiliation (short), City, Country *

University of Toronto, Toronto, Canada

University of Vienna

Your e-mail address *

abc@gmail.com

mike.schreiber@univie.ac.at

Title of your manuscript *

Provide the (draft) title of your manuscript.

A Smartphone-Based Implicit Theories Intervention for Health Behavior Change: Randomized Trial

Name of your App/Software/Intervention *

If there is a short and a long/alternate name, write the short name first and add the long name in brackets.

A Smartphone-Based Implicit Theories Interve

Evaluated Version (if any)

e.g. "V1", "Release 2017-03-01", "Version 2.0.27913"

Meine Antwort

Language(s) *

What language is the intervention/app in? If multiple languages are available, separate by comma (e.g. "English, French")

German

URL of your Intervention Website or App

e.g. a direct link to the mobile app on app in appstore (itunes, Google Play), or URL of the website. If the intervention is a DVD or hardware, you can also link to an Amazon page.

Meine Antwort

URL of an image/screenshot (optional)

Meine Antwort

Accessibility *

Can an enduser access the intervention presently?

- ○ access is free and open
- ○ access only for special usergroups, not open
- ○ access is open to everyone, but requires payment/subscription/in-app purchases
- ◉ app/intervention no longer accessible
- ○ Sonstiges:

Primary Medical Indication/Disease/Condition *

e.g. "Stress", "Diabetes", or define the target group in brackets after the condition, e.g. "Autism (Parents of children with)", "Alzheimers (Informal Caregivers of)"

Not applicable

Primary Outcomes measured in trial *

comma-separated list of primary outcomes reported in the trial

Frequency of Performing Health Promoting Be

Secondary/other outcomes

Are there any other outcomes the intervention is expected to affect?

Meine Antwort

Recommended "Dose" *

What do the instructions for users say on how often the app should be used?

- ( ) Approximately Daily
- ( ) Approximately Weekly
- ( ) Approximately Monthly
- ( ) Approximately Yearly
- ( ) "as needed"
- (x) Sonstiges: Not applicable

Approx. Percentage of Users (starters) still using the app as recommended after 3 months *

- (x) unknown / not evaluated
- ( ) 0-10%
- ( ) 11-20%
- ( ) 21-30%
- ( ) 31-40%
- ( ) 41-50%
- ( ) 51-60%
- ( ) 61-70%
- ( ) 71%-80%
- ( ) 81-90%
- ( ) 91-100%
- ( ) Sonstiges:

Overall, was the app/intervention effective? *

- ( ) yes: all primary outcomes were significantly better in intervention group vs control
- (x) partly: SOME primary outcomes were significantly better in intervention group vs control
- ( ) no statistically significant difference between control and intervention
- ( ) potentially harmful: control was significantly better than intervention in one or more outcomes
- ( ) inconclusive: more research is needed
- ( ) Sonstiges:

Article Preparation Status/Stage *

At which stage in your article preparation are you currently (at the time you fill in this form)

- ( ) not submitted yet - in early draft status
- ( ) not submitted yet - in late draft status, just before submission
- ( ) submitted to a journal but not reviewed yet
- ( ) submitted to a journal and after receiving initial reviewer comments
- (x) submitted to a journal and accepted, but not published yet
- ( ) published
- ( ) Sonstiges:

Journal *

If you already know where you will submit this paper (or if it is already submitted), please provide the journal name (if it is not JMIR, provide the journal name under "other")

- ○ not submitted yet / unclear where I will submit this
- ○ Journal of Medical Internet Research (JMIR)
- ◉ JMIR mHealth and UHealth
- ○ JMIR Serious Games
- ○ JMIR Mental Health
- ○ JMIR Public Health
- ○ JMIR Formative Research
- ○ Other JMIR sister journal
- ○ Sonstiges:

Is this a full powered effectiveness trial or a pilot/feasibility trial? *

- ○ Pilot/feasibility
- ◉ Fully powered

Manuscript tracking number *

If this is a JMIR submission, please provide the manuscript tracking number under "other" (The ms tracking number can be found in the submission acknowledgement email, or when you login as author in JMIR. If the paper is already published in JMIR, then the ms tracking number is the four-digit number at the end of the DOI, to be found at the bottom of each published article in JMIR)

- ○ no ms number (yet) / not (yet) submitted to / published in JMIR
- ◉ Sonstiges: 36578

TITLE AND ABSTRACT

1a) TITLE: Identification as a randomized trial in the title

1a) Does your paper address CONSORT item 1a? *

I.e does the title contain the phrase "Randomized Controlled Trial"? (if not, explain the reason under "other")

- ○ yes
- ◉ Sonstiges: as it is a delayed start design the title only identifies as "Randomiz

1a-i) Identify the mode of delivery in the title

Identify the mode of delivery. Preferably use “web-based” and/or “mobile” and/or “electronic game” in the title. Avoid ambiguous terms like “online”, “virtual”, “interactive”. Use “Internet-based” only if Intervention includes non-web-based Internet components (e.g. email), use “computer-based” or “electronic” only if offline products are used. Use “virtual” only in the context of “virtual reality” (3-D worlds). Use “online” only in the context of “online support groups”. Complement or substitute product names with broader terms for the class of products (such as “mobile” or “smart phone” instead of “iphone”), especially if the application runs on different platforms.

subitem not at all important

- 1 ○
- 2 ○
- 3 ○
- 4 ◉
- 5 ○

essential

Auswahl löschen

Does your paper address subitem 1a-i? *

Copy and paste relevant sections from manuscript title (include quotes in quotation marks "like this" to indicate direct quotes from your manuscript), or elaborate on this item by providing additional information not in the ms, or briefly explain why the item is not applicable/relevant for your study

Title: "A Smartphone-Based Implicit Theories Intervention for Health Behavior Change: Randomized Trial"

1a-ii) Non-web-based components or important co-interventions in title

Mention non-web-based components or important co-interventions in title, if any (e.g., “with telephone support”).

subitem not at all important

- 1 (selected)
- 2
- 3
- 4
- 5

essential

Auswahl löschen

Does your paper address subitem 1a-ii?

Copy and paste relevant sections from manuscript title (include quotes in quotation marks "like this" to indicate direct quotes from your manuscript), or elaborate on this item by providing additional information not in the ms, or briefly explain why the item is not applicable/relevant for your study

Meine Antwort

1a-iii) Primary condition or target group in the title

Mention primary condition or target group in the title, if any (e.g., “for children with Type I Diabetes”) Example: A Web-based and Mobile Intervention with Telephone Support for Children with Type I Diabetes: Randomized Controlled Trial

subitem not at all important

- 1 (selected)
- 2
- 3
- 4
- 5

essential

Auswahl löschen

Does your paper address subitem 1a-iii? *

Copy and paste relevant sections from manuscript title (include quotes in quotation marks "like this" to indicate direct quotes from your manuscript), or elaborate on this item by providing additional information not in the ms, or briefly explain why the item is not applicable/relevant for your study

Not applicable as there is no specific target group

1b) ABSTRACT: Structured summary of trial design, methods, results, and conclusions

NPT extension: Description of experimental treatment, comparator, care providers, centers, and blinding status.

1b-i) Key features/functionalities/components of the intervention and comparator in the METHODS section of the ABSTRACT

Mention key features/functionalities/components of the intervention and comparator in the abstract. If possible, also mention theories and principles used for designing the site. Keep in mind the needs of systematic reviewers and indexers by including important synonyms. (Note: Only report in the abstract what the main paper is reporting. If this information is missing from the main body of text, consider adding it)

subitem not at all important

1 ○

2 ○

3 ○

4 ◉

5 ○

essential

Auswahl löschen

Does your paper address subitem 1b-i? *

Copy and paste relevant sections from the manuscript abstract (include quotes in quotation marks "like this" to indicate direct quotes from your manuscript), or elaborate on this item by providing additional information not in the ms, or briefly explain why the item is not applicable/relevant for your study

"Methods: This 2-arm, single-blind, delayed intervention design included 149 German participants (mean age 30.58, SD 9.71 years; n=79 female). Participants were asked to report their engagement in 10 health-promoting behaviors throughout the day for 3 weeks. Participants were randomly assigned to either an early intervention group (n=72) or a delayed intervention group (n=77). The intervention materials, designed to promote an incremental theory of health, were provided to participants after 1 week (early intervention group) or 2 weeks (delayed intervention group) of baseline behavior measurement. Data for this study were collected between September 2019 and October 2019."

1b-ii) Level of human involvement in the METHODS section of the ABSTRACT

Clarify the level of human involvement in the abstract, e.g., use phrases like “fully automated” vs. “therapist/nurse/care provider/physician-assisted” (mention number and expertise of providers involved, if any). (Note: Only report in the abstract what the main paper is reporting. If this information is missing from the main body of text, consider adding it)

subitem not at all important

1 (selected)

2

3

4

5

essential

Auswahl löschen

Does your paper address subitem 1b-ii?

Copy and paste relevant sections from the manuscript abstract (include quotes in quotation marks "like this" to indicate direct quotes from your manuscript), or elaborate on this item by providing additional information not in the ms, or briefly explain why the item is not applicable/relevant for your study

Meine Antwort

1b-iii) Open vs. closed, web-based (self-assessment) vs. face-to-face assessments in the METHODS section of the ABSTRACT

Mention how participants were recruited (online vs. offline), e.g., from an open access website or from a clinic or a closed online user group (closed usergroup trial), and clarify if this was a purely web-based trial, or there were face-to-face components (as part of the intervention or for assessment). Clearly say if outcomes were self-assessed through questionnaires (as common in web-based trials). Note: In traditional offline trials, an open trial (open-label trial) is a type of clinical trial in which both the researchers and participants know which treatment is being administered. To avoid confusion, use “blinded” or “unblinded” to indicated the level of blinding instead of “open”, as “open” in web-based trials usually refers to “open access” (i.e. participants can self-enrol). (Note: Only report in the abstract what the main paper is reporting. If this information is missing from the main body of text, consider adding it)

subitem not at all important

- 1 (selected)
- 2
- 3
- 4
- 5

essential

Auswahl löschen

Does your paper address subitem 1b-iii?

Copy and paste relevant sections from the manuscript abstract (include quotes in quotation marks "like this" to indicate direct quotes from your manuscript), or elaborate on this item by providing additional information not in the ms, or briefly explain why the item is not applicable/relevant for your study

Meine Antwort

1b-iv) RESULTS section in abstract must contain use data

Report number of participants enrolled/assessed in each group, the use/uptake of the intervention (e.g., attrition/adherence metrics, use over time, number of logins etc.), in addition to primary/secondary outcomes. (Note: Only report in the abstract what the main paper is reporting. If this information is missing from the main body of text, consider adding it)

subitem not at all important

1 (selected)

2

3

4

5

essential

Auswahl löschen

Does your paper address subitem 1b-iv?

Copy and paste relevant sections from the manuscript abstract (include quotes in quotation marks "like this" to indicate direct quotes from your manuscript), or elaborate on this item by providing additional information not in the ms, or briefly explain why the item is not applicable/relevant for your study

Meine Antwort

1b-v) CONCLUSIONS/DISCUSSION in abstract for negative trials

Conclusions/Discussions in abstract for negative trials: Discuss the primary outcome - if the trial is negative (primary outcome not changed), and the intervention was not used, discuss whether negative results are attributable to lack of uptake and discuss reasons. (Note: Only report in the abstract what the main paper is reporting. If this information is missing from the main body of text, consider adding it)

subitem not at all important

1 (selected)

2

3

4

5

essential

Auswahl löschen

Does your paper address subitem 1b-v?

Copy and paste relevant sections from the manuscript abstract (include quotes in quotation marks "like this" to indicate direct quotes from your manuscript), or elaborate on this item by providing additional information not in the ms, or briefly explain why the item is not applicable/relevant for your study

Meine Antwort

INTRODUCTION

2a) In INTRODUCTION: Scientific background and explanation of rationale

2a-i) Problem and the type of system/solution

Describe the problem and the type of system/solution that is object of the study: intended as stand-alone intervention vs. incorporated in broader health care program? Intended for a particular patient population? Goals of the intervention, e.g., being more cost-effective to other interventions, replace or complement other solutions? (Note: Details about the intervention are provided in “Methods” under 5)

subitem not at all important

1 ○

2 ○

3 ◉

4 ○

5 ○

essential

Auswahl löschen

Does your paper address subitem 2a-i? *

Copy and paste relevant sections from the manuscript (include quotes in quotation marks "like this" to indicate direct quotes from your manuscript), or elaborate on this item by providing additional information not in the ms, or briefly explain why the item is not applicable/relevant for your study

" In contrast to stationary settings, the delivery of this intervention via digital technologies offers the opportunity to reach a larger audience in a sustainable manner
while minimizing implementation costs [35,36]. To increase ecological validity and to minimize recall and retrieval bias, engagement in health-promoting behaviors is measured using ecological momentary assessment in the form of daily diaries [37]. Similarly, it has been shown that stronger incremental theories of health were connected to a higher frequency of performing health-promoting behaviors in daily life measured using experience sampling (study 4) [7]. As these results were only correlational, the present research aims to provide causal insights into whether an intervention to foster incremental theories increases health behaviors in daily life. Therefore, we make the following hypothesis: Being confronted with a smartphone-based intervention to foster incremental theories of health increases the frequency of performing health-promoting behaviors in daily life."

2a-ii) Scientific background, rationale: What is known about the (type of) system

Scientific background, rationale: What is known about the (type of) system that is the object of the study (be sure to discuss the use of similar systems for other conditions/diagnoses, if appropiate), motivation for the study, i.e. what are the reasons for and what is the context for this specific study, from which stakeholder viewpoint is the study performed, potential impact of findings [2]. Briefly justify the choice of the comparator.

subitem not at all important

1 ○

2 ◉

3 ○

4 ○

5 ○

essential

Auswahl löschen

Does your paper address subitem 2a-ii? *

Copy and paste relevant sections from the manuscript (include quotes in quotation marks "like this" to indicate direct quotes from your manuscript), or elaborate on this item by providing additional information not in the ms, or briefly explain why the item is not applicable/relevant for your study

"Although correlative research shows evidence for the importance of an incremental theory of health for a healthy lifestyle, it cannot be interpreted causally. Existing experimental studies, on the other hand, generally focus only on one health behavior (eg, eating behavior). Therefore, the main purpose of this study is to investigate whether an intervention that promotes an incremental theory of health influences a variety of health-promoting behaviors. Compared with an intervention that focuses only on implicit theories in a single health domain (eg, body weight or physical activity), focusing on implicit theories of general health may serve as an efficient strategy to encourage multiple health behavior change. In contrast to stationary settings, the delivery of this intervention via digital technologies offers the opportunity to reach a larger audience in a sustainable manner while minimizing implementation costs [35,36]."

2b) In INTRODUCTION: Specific objectives or hypotheses

Does your paper address CONSORT subitem 2b? *

Copy and paste relevant sections from the manuscript (include quotes in quotation marks "like this" to indicate direct quotes from your manuscript), or elaborate on this item by providing additional information not in the ms, or briefly explain why the item is not applicable/relevant for your study

"Being confronted with a smartphone-based intervention to foster incremental theories of health increases the frequency of performing health-promoting behaviors in daily life."

METHODS

3a) Description of trial design (such as parallel, factorial) including allocation ratio

Does your paper address CONSORT subitem 3a? *

Copy and paste relevant sections from the manuscript (include quotes in quotation marks "like this" to indicate direct quotes from your manuscript), or elaborate on this item by providing additional information not in the ms, or briefly explain why the item is not applicable/relevant for your study

"We conducted a 2-arm, randomized trial to investigate whether fostering an incremental theory of health increases the frequency of performing health-promoting behaviors in daily life. The intervention was conceptualized as a delayed-start design [38,39], in which both groups received intervention material at different times. The intervention was delivered via participants' smartphones using Qualtrics (Qualtrics International) questionnaires and included that participants kept a daily diary for 3 weeks. Participants were randomly assigned (single-blind) to an early or delayed intervention group using Qualtrics' randomizer while maintaining an evenly distributed number of participants in each group (1:1 block randomization)."

3b) Important changes to methods after trial commencement (such as eligibility criteria), with reasons

Does your paper address CONSORT subitem 3b? *

Copy and paste relevant sections from the manuscript (include quotes in quotation marks "like this" to indicate direct quotes from your manuscript), or elaborate on this item by providing additional information not in the ms, or briefly explain why the item is not applicable/relevant for your study

No changes to methods after trial commencement were made

3b-i) Bug fixes, Downtimes, Content Changes

Bug fixes, Downtimes, Content Changes: ehealth systems are often dynamic systems. A description of changes to methods therefore also includes important changes made on the intervention or comparator during the trial (e.g., major bug fixes or changes in the functionality or content) (5-iii) and other “unexpected events” that may have influenced study design such as staff changes, system failures/downtimes, etc. [2].

subitem not at all important

- 1 (selected)
- 2
- 3
- 4
- 5

essential

Auswahl löschen

Does your paper address subitem 3b-i?

Copy and paste relevant sections from the manuscript (include quotes in quotation marks "like this" to indicate direct quotes from your manuscript), or elaborate on this item by providing additional information not in the ms, or briefly explain why the item is not applicable/relevant for your study

Meine Antwort

4a) Eligibility criteria for participants

Does your paper address CONSORT subitem 4a? *

Copy and paste relevant sections from the manuscript (include quotes in quotation marks "like this" to indicate direct quotes from your manuscript), or elaborate on this item by providing additional information not in the ms, or briefly explain why the item is not applicable/relevant for your study

"Eligibility criteria were a minimum age of 18 years, owning a smartphone with touch display and mobile internet access, and being able to answer daily questionnaires for 21 days."

4a-i) Computer / Internet literacy

Computer / Internet literacy is often an implicit "de facto" eligibility criterion - this should be explicitly clarified.

subitem not at all important

- 1 (selected)
- 2
- 3
- 4
- 5

essential

Auswahl löschen

Does your paper address subitem 4a-i?

Copy and paste relevant sections from the manuscript (include quotes in quotation marks "like this" to indicate direct quotes from your manuscript), or elaborate on this item by providing additional information not in the ms, or briefly explain why the item is not applicable/relevant for your study

Meine Antwort

4a-ii) Open vs. closed, web-based vs. face-to-face assessments:

Open vs. closed, web-based vs. face-to-face assessments: Mention how participants were recruited (online vs. offline), e.g., from an open access website or from a clinic, and clarify if this was a purely web-based trial, or there were face-to-face components (as part of the intervention or for assessment), i.e., to what degree got the study team to know the participant. In online-only trials, clarify if participants were quasi-anonymous and whether having multiple identities was possible or whether technical or logistical measures (e.g., cookies, email confirmation, phone calls) were used to detect/prevent these.

subitem not at all important

1 ○

2 ○

3 ◉

4 ○

5 ○

essential

Auswahl löschen

Does your paper address subitem 4a-ii? *

Copy and paste relevant sections from the manuscript (include quotes in quotation marks "like this" to indicate direct quotes from your manuscript), or elaborate on this item by providing additional information not in the ms, or briefly explain why the item is not applicable/relevant for your study

"Participants were recruited via the institutes' participant pool and social media postings."

"Over the course of 3 weeks (21 days), participants received daily invitations to complete a short questionnaire via texting distributed via SurveySignal [40]."

4a-iii) Information giving during recruitment

Information given during recruitment. Specify how participants were briefed for recruitment and in the informed consent procedures (e.g., publish the informed consent documentation as appendix, see also item X26), as this information may have an effect on user self-selection, user expectation and may also bias results.

subitem not at all important

- 1 (selected)
- 2
- 3
- 4
- 5

essential

Auswahl löschen

Does your paper address subitem 4a-iii?

Copy and paste relevant sections from the manuscript (include quotes in quotation marks "like this" to indicate direct quotes from your manuscript), or elaborate on this item by providing additional information not in the ms, or briefly explain why the item is not applicable/relevant for your study

Meine Antwort

4b) Settings and locations where the data were collected

Does your paper address CONSORT subitem 4b? *

Copy and paste relevant sections from the manuscript (include quotes in quotation marks "like this" to indicate direct quotes from your manuscript), or elaborate on this item by providing additional information not in the ms, or briefly explain why the item is not applicable/relevant for your study

"Data for this study were collected between September 2019 and October 2019."
"The intervention was delivered via participants' smartphones using Qualtrics (Qualtrics International) questionnaires and included that participants kept a daily diary for 3 weeks."

4b-i) Report if outcomes were (self-)assessed through online questionnaires

Clearly report if outcomes were (self-)assessed through online questionnaires (as common in web-based trials) or otherwise.

subitem not at all important

1 ○

2 ○

3 ○

4 ○

5 ○

essential

Does your paper address subitem 4b-i? *

Copy and paste relevant sections from the manuscript (include quotes in quotation marks "like this" to indicate direct quotes from your manuscript), or elaborate on this item by providing additional information not in the ms, or briefly explain why the item is not applicable/relevant for your study

"Over the course of 3 weeks (21 days), participants received daily invitations to complete a short questionnaire via texting distributed via SurveySignal [40]."

4b-ii) Report how institutional affiliations are displayed

Report how institutional affiliations are displayed to potential participants [on ehealth media], as affiliations with prestigious hospitals or universities may affect volunteer rates, use, and reactions with regards to an intervention.(Not a required item – describe only if this may bias results)

subitem not at all important

- 1 (selected)
- 2
- 3
- 4
- 5

essential

Auswahl löschen

Does your paper address subitem 4b-ii?

Copy and paste relevant sections from the manuscript (include quotes in quotation marks "like this" to indicate direct quotes from your manuscript), or elaborate on this item by providing additional information not in the ms, or briefly explain why the item is not applicable/relevant for your study

Meine Antwort

5) The interventions for each group with sufficient details to allow replication, including how and when they were actually administered

5-i) Mention names, credential, affiliations of the developers, sponsors, and owners

Mention names, credential, affiliations of the developers, sponsors, and owners [6] (if authors/evaluators are owners or developer of the software, this needs to be declared in a “Conflict of interest” section or mentioned elsewhere in the manuscript).

subitem not at all important

1 ○

2 ○

3 ○

4 ◉

5 ○

essential

Auswahl löschen

Does your paper address subitem 5-i?

Copy and paste relevant sections from the manuscript (include quotes in quotation marks "like this" to indicate direct quotes from your manuscript), or elaborate on this item by providing additional information not in the ms, or briefly explain why the item is not applicable/relevant for your study

"Participants received a link to the intervention materials on their smartphones either after 7 (early intervention group) or 14 days (delayed intervention group) of baseline measurement. The links to view the intervention materials were sent out at 8 AM on the specified day (day 8 or 15), and the individual links were available until 8 PM on that day. Like other interventions to promote incremental theories [12,20,22], the intervention materials consisted of informative, exemplary, and reflective components. More precisely, the intervention materials included(1) a (fictitious) newspaper article that described health as mainly influenced by lifestyle and engagement in health-promoting behavior [7], (2) three fictitious blog posts in which individuals reported positive health changes, (3) an essay priming in which participants were asked to describe health changes in their lives, and (4) an article that focused on the benefits of beliefs in changeability in other domains. The materials are available in the OSF repository:

Schreiber M, Dohle S. Implicit Theories Intervention for Multiple Health Behavior Change. 2019. URL: https://osf.io/y7un5/ [accessed 2023-05-16]"

5-ii) Describe the history/development process

Describe the history/development process of the application and previous formative evaluations (e.g., focus groups, usability testing), as these will have an impact on adoption/use rates and help with interpreting results.

subitem not at all important

1 (selected)

2

3

4

5

essential

Auswahl löschen

Does your paper address subitem 5-ii?

Copy and paste relevant sections from the manuscript (include quotes in quotation marks "like this" to indicate direct quotes from your manuscript), or elaborate on this item by providing additional information not in the ms, or briefly explain why the item is not applicable/relevant for your study

Meine Antwort

5-iii) Revisions and updating

Revisions and updating. Clearly mention the date and/or version number of the application/intervention (and comparator, if applicable) evaluated, or describe whether the intervention underwent major changes during the evaluation process, or whether the development and/or content was "frozen" during the trial. Describe dynamic components such as news feeds or changing content which may have an impact on the replicability of the intervention (for unexpected events see item 3b).

subitem not at all important

- 1 (selected)
- 2
- 3
- 4
- 5

essential

Auswahl löschen

Does your paper address subitem 5-iii?

Copy and paste relevant sections from the manuscript (include quotes in quotation marks "like this" to indicate direct quotes from your manuscript), or elaborate on this item by providing additional information not in the ms, or briefly explain why the item is not applicable/relevant for your study

Meine Antwort

5-iv) Quality assurance methods

Provide information on quality assurance methods to ensure accuracy and quality of information provided [1], if applicable.

subitem not at all important

1 (selected)

2

3

4

5

essential

Auswahl löschen

Does your paper address subitem 5-iv?

Copy and paste relevant sections from the manuscript (include quotes in quotation marks "like this" to indicate direct quotes from your manuscript), or elaborate on this item by providing additional information not in the ms, or briefly explain why the item is not applicable/relevant for your study

Meine Antwort

5-v) Ensure replicability by publishing the source code, and/or providing screenshots/screen-capture video, and/or providing flowcharts of the algorithms used

Ensure replicability by publishing the source code, and/or providing screenshots/screen-capture video, and/or providing flowcharts of the algorithms used. Replicability (i.e., other researchers should in principle be able to replicate the study) is a hallmark of scientific reporting.

subitem not at all important

1 (x)

2 ( )

3 ( )

4 ( )

5 ( )

essential

Auswahl löschen

Does your paper address subitem 5-v?

Copy and paste relevant sections from the manuscript (include quotes in quotation marks "like this" to indicate direct quotes from your manuscript), or elaborate on this item by providing additional information not in the ms, or briefly explain why the item is not applicable/relevant for your study

Meine Antwort

5-vi) Digital preservation

Digital preservation: Provide the URL of the application, but as the intervention is likely to change or disappear over the course of the years; also make sure the intervention is archived (Internet Archive, webcitation.org, and/or publishing the source code or screenshots/videos alongside the article). As pages behind login screens cannot be archived, consider creating demo pages which are accessible without login.

subitem not at all important

- 1 (selected)
- 2
- 3
- 4
- 5

essential

Auswahl löschen

Does your paper address subitem 5-vi?

Copy and paste relevant sections from the manuscript (include quotes in quotation marks "like this" to indicate direct quotes from your manuscript), or elaborate on this item by providing additional information not in the ms, or briefly explain why the item is not applicable/relevant for your study

Meine Antwort

5-vii) Access

Access: Describe how participants accessed the application, in what setting/context, if they had to pay (or were paid) or not, whether they had to be a member of specific group. If known, describe how participants obtained “access to the platform and Internet” [1]. To ensure access for editors/reviewers/readers, consider to provide a “backdoor” login account or demo mode for reviewers/readers to explore the application (also important for archiving purposes, see vi).

subitem not at all important

1 ○

2 ○

3 ◉

4 ○

5 ○

essential

Auswahl löschen

Does your paper address subitem 5-vii? *

Copy and paste relevant sections from the manuscript (include quotes in quotation marks "like this" to indicate direct quotes from your manuscript), or elaborate on this item by providing additional information not in the ms, or briefly explain why the item is not applicable/relevant for your study

"Participants were recruited via the institutes’ participant pool and social media postings."

"The intervention was delivered via participants’ smartphones using Qualtrics (Qualtrics International) questionnaires and included that participants kept a daily diary for 3 weeks."

"Over the course of 3 weeks (21 days), participants received daily invitations to complete a short questionnaire via texting distributed via SurveySignal [40]."

5-viii) Mode of delivery, features/functionalities/components of the intervention and comparator, and the theoretical framework

Describe mode of delivery, features/functionalities/components of the intervention and comparator, and the theoretical framework [6] used to design them (instructional strategy [1], behaviour change techniques, persuasive features, etc., see e.g., [7, 8] for terminology). This includes an in-depth description of the content (including where it is coming from and who developed it) [1],” whether [and how] it is tailored to individual circumstances and allows users to track their progress and receive feedback” [6]. This also includes a description of communication delivery channels and – if computer-mediated communication is a component – whether communication was synchronous or asynchronous [6]. It also includes information on presentation strategies [1], including page design principles, average amount of text on pages, presence of hyperlinks to other resources, etc. [1].

subitem not at all important

1 ○

2 ○

3 ○

4 ○

5 ○

essential

Does your paper address subitem 5-viii? *

Copy and paste relevant sections from the manuscript (include quotes in quotation marks "like this" to indicate direct quotes from your manuscript), or elaborate on this item by providing additional information not in the ms, or briefly explain why the item is not applicable/relevant for your study

"Participants received a link to the intervention materials on their smartphones either after 7 (early intervention group) or 14 days (delayed intervention group) of baseline measurement. The links to view the intervention materials were sent out at 8 AM on the specified day (day 8 or 15), and the individual links were available until 8 PM on that day. Like other interventions to promote incremental theories [12,20,22], the intervention materials consisted of informative, exemplary, and reflective components. More precisely, the intervention materials included(1) a (fictitious) newspaper article that described health as mainly influenced by lifestyle and engagement in health-promoting behavior [7], (2) three fictitious blog posts in which individuals reported positive health changes, (3) an essay priming in which participants were asked to describe health changes in their lives, and (4) an article that focused on the benefits of beliefs in changeability in other domains. The materials are available in the OSF repository:

Schreiber M, Dohle S. Implicit Theories Intervention for Multiple Health Behavior Change. 2019. URL: https://osf.io/y7un5/ [accessed 2023-05-16]"

5-ix) Describe use parameters

Describe use parameters (e.g., intended "doses" and optimal timing for use). Clarify what instructions or recommendations were given to the user, e.g., regarding timing, frequency, heaviness of use, if any, or was the intervention used ad libitum.

subitem not at all important

- 1 (selected)
- 2
- 3
- 4
- 5

essential

Auswahl löschen

Does your paper address subitem 5-ix?

Copy and paste relevant sections from the manuscript (include quotes in quotation marks "like this" to indicate direct quotes from your manuscript), or elaborate on this item by providing additional information not in the ms, or briefly explain why the item is not applicable/relevant for your study

Meine Antwort

5-x) Clarify the level of human involvement

Clarify the level of human involvement (care providers or health professionals, also technical assistance) in the e-intervention or as co-intervention (detail number and expertise of professionals involved, if any, as well as “type of assistance offered, the timing and frequency of the support, how it is initiated, and the medium by which the assistance is delivered”. It may be necessary to distinguish between the level of human involvement required for the trial, and the level of human involvement required for a routine application outside of a RCT setting (discuss under item 21 – generalizability).

subitem not at all important

- 1 (selected)
- 2
- 3
- 4
- 5

essential

Auswahl löschen

Does your paper address subitem 5-x?

Copy and paste relevant sections from the manuscript (include quotes in quotation marks "like this" to indicate direct quotes from your manuscript), or elaborate on this item by providing additional information not in the ms, or briefly explain why the item is not applicable/relevant for your study

Meine Antwort

5-xi) Report any prompts/reminders used

Report any prompts/reminders used: Clarify if there were prompts (letters, emails, phone calls, SMS) to use the application, what triggered them, frequency etc. It may be necessary to distinguish between the level of prompts/reminders required for the trial, and the level of prompts/reminders for a routine application outside of a RCT setting (discuss under item 21 – generalizability).

subitem not at all important

1 ○

2 ○

3 ◉

4 ○

5 ○

essential

Auswahl löschen

Does your paper address subitem 5-xi? *

Copy and paste relevant sections from the manuscript (include quotes in quotation marks "like this" to indicate direct quotes from your manuscript), or elaborate on this item by providing additional information not in the ms, or briefly explain why the item is not applicable/relevant for your study

"Over the course of 3 weeks (21 days), participants received daily invitations to complete a short questionnaire via texting distributed via SurveySignal [40]. Participants received the invitations daily at 8 PM and had to respond within 4 hours."

No further reminders or prompts were used.

5-xii) Describe any co-interventions (incl. training/support)

Describe any co-interventions (incl. training/support): Clearly state any interventions that are provided in addition to the targeted eHealth intervention, as ehealth intervention may not be designed as stand-alone intervention. This includes training sessions and support [1]. It may be necessary to distinguish between the level of training required for the trial, and the level of training for a routine application outside of a RCT setting (discuss under item 21 – generalizability.

subitem not at all important

- 1 (selected)
- 2
- 3
- 4
- 5

essential

Auswahl löschen

Does your paper address subitem 5-xii? *

Copy and paste relevant sections from the manuscript (include quotes in quotation marks "like this" to indicate direct quotes from your manuscript), or elaborate on this item by providing additional information not in the ms, or briefly explain why the item is not applicable/relevant for your study

No co-interventions were used.

6a) Completely defined pre-specified primary and secondary outcome measures, including how and when they were assessed

Does your paper address CONSORT subitem 6a? *

Copy and paste relevant sections from the manuscript (include quotes in quotation marks "like this" to indicate direct quotes from your manuscript), or elaborate on this item by providing additional information not in the ms, or briefly explain why the item is not applicable/relevant for your study

"In the daily diaries, participants were asked every day whether they performed 10 health-promoting behaviors throughout the respective day (see Textbox 1; 0=no, 1=yes). We measured only behaviors (1) that could be performed during a regular day, (2) that were based on national recommendations from public health authorities (eg, Federal Centre for Health Education), and (3) that showed no ceiling or floor effect regarding the frequency of performing these behaviors, determined in a pretest (n=325). Concerning the latter, we did not include behaviors such as brushing one's teeth or washing one's hands because the pretest showed that almost all participants conducted these behaviors daily. The sum of performed health-promoting behaviors per day served as the primary outcome measure.

6a-i) Online questionnaires: describe if they were validated for online use and apply CHERRIES items to describe how the questionnaires were designed/deployed

If outcomes were obtained through online questionnaires, describe if they were validated for online use and apply CHERRIES items to describe how the questionnaires were designed/deployed [9].

subitem not at all important

- 1 (selected)
- 2
- 3
- 4
- 5

essential

Auswahl löschen

Does your paper address subitem 6a-i?

Copy and paste relevant sections from manuscript text

Meine Antwort

6a-ii) Describe whether and how “use” (including intensity of use/dosage) was defined/measured/monitored

Describe whether and how “use” (including intensity of use/dosage) was defined/measured/monitored (logins, logfile analysis, etc.). Use/adoption metrics are important process outcomes that should be reported in any ehealth trial.

subitem not at all important

- 1 (selected)
- 2
- 3
- 4
- 5

essential

Auswahl löschen

Does your paper address subitem 6a-ii?

Copy and paste relevant sections from manuscript text

Meine Antwort

6a-iii) Describe whether, how, and when qualitative feedback from participants was obtained

Describe whether, how, and when qualitative feedback from participants was obtained (e.g., through emails, feedback forms, interviews, focus groups).

subitem not at all important

1 (selected)

2

3

4

5

essential

Auswahl löschen

Does your paper address subitem 6a-iii?

Copy and paste relevant sections from manuscript text

Meine Antwort

6b) Any changes to trial outcomes after the trial commenced, with reasons

Does your paper address CONSORT subitem 6b? *

Copy and paste relevant sections from the manuscript (include quotes in quotation marks "like this" to indicate direct quotes from your manuscript), or elaborate on this item by providing additional information not in the ms, or briefly explain why the item is not applicable/relevant for your study

No changes to trial outcomes after the trial commenced were made

7a) How sample size was determined

NPT: When applicable, details of whether and how the clustering by care provides or centers was addressed

7a-i) Describe whether and how expected attrition was taken into account when calculating the sample size

Describe whether and how expected attrition was taken into account when calculating the sample size.

subitem not at all important

1 (selected)

2

3

4

5

essential

Auswahl löschen

Does your paper address subitem 7a-i?

Copy and paste relevant sections from manuscript title (include quotes in quotation marks "like this" to indicate direct quotes from your manuscript), or elaborate on this item by providing additional information not in the ms, or briefly explain why the item is not applicable/relevant for your study

Meine Antwort

7b) When applicable, explanation of any interim analyses and stopping guidelines

Does your paper address CONSORT subitem 7b? *

Copy and paste relevant sections from the manuscript (include quotes in quotation marks "like this" to indicate direct quotes from your manuscript), or elaborate on this item by providing additional information not in the ms, or briefly explain why the item is not applicable/relevant for your study

"Because we also planned to run multilevel models, a total sample size of 120 was targeted to increase the probability of achieving model convergence. As described further in the Results section, main analyses were performed using data from 149 participants."

No further interim analyses or stopping guidelines were used.

8a) Method used to generate the random allocation sequence

NPT: When applicable, how care providers were allocated to each trial group

Does your paper address CONSORT subitem 8a? *

Copy and paste relevant sections from the manuscript (include quotes in quotation marks "like this" to indicate direct quotes from your manuscript), or elaborate on this item by providing additional information not in the ms, or briefly explain why the item is not applicable/relevant for your study

"Participants were randomly assigned (single-blind) to an early or delayed intervention group using Qualtrics' randomizer while maintaining an evenly distributed number of participants in each group (1:1 block randomization)."

8b) Type of randomisation; details of any restriction (such as blocking and block size)

Does your paper address CONSORT subitem 8b? *

Copy and paste relevant sections from the manuscript (include quotes in quotation marks "like this" to indicate direct quotes from your manuscript), or elaborate on this item by providing additional information not in the ms, or briefly explain why the item is not applicable/relevant for your study

"Participants were randomly assigned (single-blind) to an early or delayed intervention group using Qualtrics' randomizer while maintaining an evenly distributed number of participants in each group (1:1 block randomization)."

9) Mechanism used to implement the random allocation sequence (such as sequentially numbered containers), describing any steps taken to conceal the sequence until interventions were assigned

Does your paper address CONSORT subitem 9? *

Copy and paste relevant sections from the manuscript (include quotes in quotation marks "like this" to indicate direct quotes from your manuscript), or elaborate on this item by providing additional information not in the ms, or briefly explain why the item is not applicable/relevant for your study

Randomization via Qualtrics.

10) Who generated the random allocation sequence, who enrolled participants, and who assigned participants to interventions

Does your paper address CONSORT subitem 10? *

Copy and paste relevant sections from the manuscript (include quotes in quotation marks "like this" to indicate direct quotes from your manuscript), or elaborate on this item by providing additional information not in the ms, or briefly explain why the item is not applicable/relevant for your study

Random Allocation Sequence was generated via Qualtrics.

11a) If done, who was blinded after assignment to interventions (for example, participants, care providers, those assessing outcomes) and how

NPT: Whether or not administering co-interventions were blinded to group assignment

11a-i) Specify who was blinded, and who wasn't

Specify who was blinded, and who wasn't. Usually, in web-based trials it is not possible to blind the participants [1, 3] (this should be clearly acknowledged), but it may be possible to blind outcome assessors, those doing data analysis or those administering co-interventions (if any).

subitem not at all important

1 ○

2 ○

3 ○

4 ◉

5 ○

essential

Auswahl löschen

Does your paper address subitem 11a-i? *

Copy and paste relevant sections from the manuscript (include quotes in quotation marks "like this" to indicate direct quotes from your manuscript), or elaborate on this item by providing additional information not in the ms, or briefly explain why the item is not applicable/relevant for your study

"Participants were randomly assigned (single-blind) to an early or delayed intervention group"

11a-ii) Discuss e.g., whether participants knew which intervention was the "intervention of interest" and which one was the "comparator"

Informed consent procedures (4a-ii) can create biases and certain expectations - discuss e.g., whether participants knew which intervention was the "intervention of interest" and which one was the "comparator".

subitem not at all important

1 (selected)

2

3

4

5

essential

Auswahl löschen

Does your paper address subitem 11a-ii?

Copy and paste relevant sections from the manuscript (include quotes in quotation marks "like this" to indicate direct quotes from your manuscript), or elaborate on this item by providing additional information not in the ms, or briefly explain why the item is not applicable/relevant for your study

Meine Antwort

11b) If relevant, description of the similarity of interventions

(this item is usually not relevant for ehealth trials as it refers to similarity of a placebo or sham intervention to a active medication/intervention)

Does your paper address CONSORT subitem 11b? *

Copy and paste relevant sections from the manuscript (include quotes in quotation marks "like this" to indicate direct quotes from your manuscript), or elaborate on this item by providing additional information not in the ms, or briefly explain why the item is not applicable/relevant for your study

"We chose the delayed-start design as it allows testing for intervention effects between and within both intervention arms [38,39] and helps to disentangle the effects of the intervention itself and the self-monitoring due to the daily diaries."

Both intervention arms received the intervention materials at different times.

12a) Statistical methods used to compare groups for primary and secondary outcomes

NPT: When applicable, details of whether and how the clustering by care providers or centers was addressed

Does your paper address CONSORT subitem 12a? *

Copy and paste relevant sections from the manuscript (include quotes in quotation marks "like this" to indicate direct quotes from your manuscript), or elaborate on this item by providing additional information not in the ms, or briefly explain why the item is not applicable/relevant for your study

"Based on the preregistration [41], a mixed ANOVA was conducted to test whether the intervention increased the frequency of performing health-promoting behaviors on a weekly level. [...] Therefore, the preregistered multilevel models were performed to test whether the intervention increased the frequency of performing health-promoting behaviors on a daily level. Day was treated as the level 1 unit and participant as the level 2 unit. Intervention status (0=before intervention, 1=after intervention) served as the level 1 predictor, whereas the number of performed health-promoting behaviors served as the level 1–dependent variable."

12a-i) Imputation techniques to deal with attrition / missing values

Imputation techniques to deal with attrition / missing values: Not all participants will use the intervention/comparator as intended and attrition is typically high in ehealth trials. Specify how participants who did not use the application or dropped out from the trial were treated in the statistical analysis (a complete case analysis is strongly discouraged, and simple imputation techniques such as LOCF may also be problematic [4]).

subitem not at all important

- 1 (selected)
- 2
- 3
- 4
- 5

essential

Auswahl löschen

Does your paper address subitem 12a-i? *

Copy and paste relevant sections from the manuscript (include quotes in quotation marks "like this" to indicate direct quotes from your manuscript), or elaborate on this item by providing additional information not in the ms, or briefly explain why the item is not applicable/relevant for your study

No imputation methods were used.

12b) Methods for additional analyses, such as subgroup analyses and adjusted analyses

Does your paper address CONSORT subitem 12b? *

Copy and paste relevant sections from the manuscript (include quotes in quotation marks "like this" to indicate direct quotes from your manuscript), or elaborate on this item by providing additional information not in the ms, or briefly explain why the item is not applicable/relevant for your study

"Additional analyses revealed that participants did also report a stronger incremental theory in the follow-up questionnaire (mean 5.42, SE 0.08) compared with the entry questionnaire (mean 5.24, SE 0.08; t137=2.42, SE 0.07; P=.02; 95% CI 0.03-0.32; d=0.20). Furthermore, participants reported a stronger internal health locus of control in the follow-up questionnaire (mean 3.92, SE 0.06) compared with the entry questionnaire (mean 3.78, SE 0.06; t137=3.17, SE 0.04; P=.002; 95% CI 0.05-0.23; d=0.28). To test whether our intervention also led to an increase in health-promoting behaviors when controlling for the change in internal health locus of control, we performed additional multilevel models, including the change in internal health locus of control as the level 2 predictor. Table 5 shows that these robustness checks led to the same conclusions as the multilevel models described in the previous section."

X26) REB/IRB Approval and Ethical Considerations [recommended as subheading under "Methods"] (not a CONSORT item)

X26-i) Comment on ethics committee approval

subitem not at all important

- 1 ○
- 2 ○
- 3 ○
- 4 ◉
- 5 ○

essential

Auswahl löschen

Does your paper address subitem X26-i?

Copy and paste relevant sections from the manuscript (include quotes in quotation marks "like this" to indicate direct quotes from your manuscript), or elaborate on this item by providing additional information not in the ms, or briefly explain why the item is not applicable/relevant for your study

"The study was approved by the faculty's ethics commission (ID MSHF0047)."

x26-ii) Outline informed consent procedures

Outline informed consent procedures e.g., if consent was obtained offline or online (how? Checkbox, etc.?), and what information was provided (see 4a-ii). See [6] for some items to be included in informed consent documents.

subitem not at all important

- 1 (selected)
- 2
- 3
- 4
- 5

essential

Auswahl löschen

Does your paper address subitem X26-ii?

Copy and paste relevant sections from the manuscript (include quotes in quotation marks "like this" to indicate direct quotes from your manuscript), or elaborate on this item by providing additional information not in the ms, or briefly explain why the item is not applicable/relevant for your study

Meine Antwort

X26-iii) Safety and security procedures

Safety and security procedures, incl. privacy considerations, and any steps taken to reduce the likelihood or detection of harm (e.g., education and training, availability of a hotline)

subitem not at all important

1 (selected)

2

3

4

5

essential

Auswahl löschen

Does your paper address subitem X26-iii?

Copy and paste relevant sections from the manuscript (include quotes in quotation marks "like this" to indicate direct quotes from your manuscript), or elaborate on this item by providing additional information not in the ms, or briefly explain why the item is not applicable/relevant for your study

Meine Antwort

RESULTS

13a) For each group, the numbers of participants who were randomly assigned, received intended treatment, and were analysed for the primary outcome

NPT: The number of care providers or centers performing the intervention in each group and the number of patients treated by each care provider in each center

Does your paper address CONSORT subitem 13a? *

Copy and paste relevant sections from the manuscript (include quotes in quotation marks "like this" to indicate direct quotes from your manuscript), or elaborate on this item by providing additional information not in the ms, or briefly explain why the item is not applicable/relevant for your study

See Figure 1. CONSORT (Consolidated Standards of Reporting Trials) flow diagram.

13b) For each group, losses and exclusions after randomisation, together with reasons

Does your paper address CONSORT subitem 13b? (NOTE: Preferably, this is shown in a CONSORT flow diagram) *

Copy and paste relevant sections from the manuscript (include quotes in quotation marks "like this" to indicate direct quotes from your manuscript), or elaborate on this item by providing additional information not in the ms, or briefly explain why the item is not applicable/relevant for your study

See Figure 1. CONSORT (Consolidated Standards of Reporting Trials) flow diagram.

13b-i) Attrition diagram

Strongly recommended: An attrition diagram (e.g., proportion of participants still logging in or using the intervention/comparator in each group plotted over time, similar to a survival curve) or other figures or tables demonstrating usage/dose/engagement.

subitem not at all important

1 (selected)

2

3

4

5

essential

Auswahl löschen

Does your paper address subitem 13b-i?

Copy and paste relevant sections from the manuscript or cite the figure number if applicable (include quotes in quotation marks "like this" to indicate direct quotes from your manuscript), or elaborate on this item by providing additional information not in the ms, or briefly explain why the item is not applicable/relevant for your study

Meine Antwort

14a) Dates defining the periods of recruitment and follow-up

Does your paper address CONSORT subitem 14a? *

Copy and paste relevant sections from the manuscript (include quotes in quotation marks "like this" to indicate direct quotes from your manuscript), or elaborate on this item by providing additional information not in the ms, or briefly explain why the item is not applicable/relevant for your study

"Data collection for this study was performed between September 13, 2019, and October 10, 2019."
"After 21 days, we invited participants to participate in a follow-up questionnaire"

14a-i) Indicate if critical “secular events” fell into the study period

Indicate if critical “secular events” fell into the study period, e.g., significant changes in Internet resources available or “changes in computer hardware or Internet delivery resources”

subitem not at all important

- 1 (selected)
- 2
- 3
- 4
- 5

essential

Auswahl löschen

Does your paper address subitem 14a-i?

Copy and paste relevant sections from the manuscript (include quotes in quotation marks "like this" to indicate direct quotes from your manuscript), or elaborate on this item by providing additional information not in the ms, or briefly explain why the item is not applicable/relevant for your study

Meine Antwort

14b) Why the trial ended or was stopped (early)

Does your paper address CONSORT subitem 14b? *

Copy and paste relevant sections from the manuscript (include quotes in quotation marks "like this" to indicate direct quotes from your manuscript), or elaborate on this item by providing additional information not in the ms, or briefly explain why the item is not applicable/relevant for your study

We did not stop the trial earlier as planned

15) A table showing baseline demographic and clinical characteristics for each group

NPT: When applicable, a description of care providers (case volume, qualification, expertise, etc.) and centers (volume) in each group

Does your paper address CONSORT subitem 15? *

Copy and paste relevant sections from the manuscript (include quotes in quotation marks "like this" to indicate direct quotes from your manuscript), or elaborate on this item by providing additional information not in the ms, or briefly explain why the item is not applicable/relevant for your study

See Table 3. Baseline characteristics of participants in total and by intervention group (n=149).

15-i) Report demographics associated with digital divide issues

In ehealth trials it is particularly important to report demographics associated with digital divide issues, such as age, education, gender, social-economic status, computer/Internet/ehealth literacy of the participants, if known.

subitem not at all important

1 ○

2 ○

3 ◉

4 ○

5 ○

essential

Auswahl löschen

Does your paper address subitem 15-i? *

Copy and paste relevant sections from the manuscript (include quotes in quotation marks "like this" to indicate direct quotes from your manuscript), or elaborate on this item by providing additional information not in the ms, or briefly explain why the item is not applicable/relevant for your study

Table 3 inlcuded information on Age, Height, Weight, BMI, Gender, Education Level, Occupation

16) For each group, number of participants (denominator) included in each analysis and whether the analysis was by original assigned groups

16-i) Report multiple “denominators” and provide definitions

Report multiple “denominators” and provide definitions: Report N’s (and effect sizes) “across a range of study participation [and use] thresholds” [1], e.g., N exposed, N consented, N used more than x times, N used more than y weeks, N participants “used” the intervention/comparator at specific pre-defined time points of interest (in absolute and relative numbers per group). Always clearly define “use” of the intervention.

subitem not at all important

- 1 (selected)
- 2
- 3
- 4
- 5

essential

Auswahl löschen

Does your paper address subitem 16-i? *

Copy and paste relevant sections from the manuscript (include quotes in quotation marks "like this" to indicate direct quotes from your manuscript), or elaborate on this item by providing additional information not in the ms, or briefly explain why the item is not applicable/relevant for your study

Information is available in Table 3

16-ii) Primary analysis should be intent-to-treat

Primary analysis should be intent-to-treat, secondary analyses could include comparing only “users”, with the appropriate caveats that this is no longer a randomized sample (see 18-i).

subitem not at all important

1 (selected)

2

3

4

5

essential

Auswahl löschen

Does your paper address subitem 16-ii?

Copy and paste relevant sections from the manuscript (include quotes in quotation marks "like this" to indicate direct quotes from your manuscript), or elaborate on this item by providing additional information not in the ms, or briefly explain why the item is not applicable/relevant for your study

Meine Antwort

17a) For each primary and secondary outcome, results for each group, and the estimated effect size and its precision (such as 95% confidence interval)

Does your paper address CONSORT subitem 17a? *

Copy and paste relevant sections from the manuscript (include quotes in quotation marks "like this" to indicate direct quotes from your manuscript), or elaborate on this item by providing additional information not in the ms, or briefly explain why the item is not applicable/relevant for your study

Effect sizes are included.

17a-i) Presentation of process outcomes such as metrics of use and intensity of use

In addition to primary/secondary (clinical) outcomes, the presentation of process outcomes such as metrics of use and intensity of use (dose, exposure) and their operational definitions is critical. This does not only refer to metrics of attrition (13-b) (often a binary variable), but also to more continuous exposure metrics such as “average session length”. These must be accompanied by a technical description how a metric like a “session” is defined (e.g., timeout after idle time) [1] (report under item 6a).

subitem not at all important

1 (selected)

2

3

4

5

essential

Auswahl löschen

Does your paper address subitem 17a-i?

Copy and paste relevant sections from the manuscript (include quotes in quotation marks "like this" to indicate direct quotes from your manuscript), or elaborate on this item by providing additional information not in the ms, or briefly explain why the item is not applicable/relevant for your study

Meine Antwort

17b) For binary outcomes, presentation of both absolute and relative effect sizes is recommended

Does your paper address CONSORT subitem 17b? *

Copy and paste relevant sections from the manuscript (include quotes in quotation marks "like this" to indicate direct quotes from your manuscript), or elaborate on this item by providing additional information not in the ms, or briefly explain why the item is not applicable/relevant for your study

Not applicable as no binary outcomes were of interest.

18) Results of any other analyses performed, including subgroup analyses and adjusted analyses, distinguishing pre-specified from exploratory

Does your paper address CONSORT subitem 18? *

Copy and paste relevant sections from the manuscript (include quotes in quotation marks "like this" to indicate direct quotes from your manuscript), or elaborate on this item by providing additional information not in the ms, or briefly explain why the item is not applicable/relevant for your study

"Additional analyses revealed that participants did also report a stronger incremental theory in the follow-up questionnaire (mean 5.42, SE 0.08) compared with the entry questionnaire (mean 5.24, SE 0.08; t_{137}=2.42, SE 0.07; P=.02; 95% CI 0.03-0.32; d=0.20). Furthermore, participants reported a stronger internal health locus of control in the follow-up questionnaire (mean 3.92, SE 0.06) compared with the entry questionnaire (mean 3.78, SE 0.06; t_{137}=3.17, SE 0.04; P=.002; 95% CI 0.05-0.23; d=0.28). To test whether our intervention also led to an increase in health-promoting behaviors when controlling for the change in internal health locus of control, we performed additional multilevel models, including the change in internal health locus of control as the level 2 predictor. Table 5 shows that these robustness checks led to the same conclusions as the multilevel models described in the previous section."

18-i) Subgroup analysis of comparing only users

A subgroup analysis of comparing only users is not uncommon in ehealth trials, but if done, it must be stressed that this is a self-selected sample and no longer an unbiased sample from a randomized trial (see 16-iii).

subitem not at all important

- 1 (selected)
- 2
- 3
- 4
- 5

essential

Auswahl löschen

Does your paper address subitem 18-i?

Copy and paste relevant sections from the manuscript (include quotes in quotation marks "like this" to indicate direct quotes from your manuscript), or elaborate on this item by providing additional information not in the ms, or briefly explain why the item is not applicable/relevant for your study

Meine Antwort

19) All important harms or unintended effects in each group

(for specific guidance see CONSORT for harms)

Does your paper address CONSORT subitem 19? *

Copy and paste relevant sections from the manuscript (include quotes in quotation marks "like this" to indicate direct quotes from your manuscript), or elaborate on this item by providing additional information not in the ms, or briefly explain why the item is not applicable/relevant for your study

"This study is in line with the majority of research showing that a stronger incremental theory leads to beneficial outcomes [5]. However, holding an entity theory can be instrumental under specific circumstances. A stronger incremental theory of health not only implies that one's health can improve but also means that one's health might worsen. For this reason, an incremental theory would be less adaptive when a prevention focus is present [52,53], that is, when one is trying to conserve a given health status. For individuals being confronted with the process of aging or suffering from long-lasting diseases, it may be more beneficial to believe in the stability of health."

19-i) Include privacy breaches, technical problems

Include privacy breaches, technical problems. This does not only include physical "harm" to participants, but also incidents such as perceived or real privacy breaches [1], technical problems, and other unexpected/unintended incidents. "Unintended effects" also includes unintended positive effects [2].

subitem not at all important

- 1 (selected)
- 2
- 3
- 4
- 5

essential

Auswahl löschen

Does your paper address subitem 19-i?

Copy and paste relevant sections from the manuscript (include quotes in quotation marks "like this" to indicate direct quotes from your manuscript), or elaborate on this item by providing additional information not in the ms, or briefly explain why the item is not applicable/relevant for your study

Meine Antwort

19-ii) Include qualitative feedback from participants or observations from staff/researchers

Include qualitative feedback from participants or observations from staff/researchers, if available, on strengths and shortcomings of the application, especially if they point to unintended/unexpected effects or uses. This includes (if available) reasons for why people did or did not use the application as intended by the developers.

subitem not at all important

- 1 (selected)
- 2
- 3
- 4
- 5

essential

Auswahl löschen

Does your paper address subitem 19-ii?

Copy and paste relevant sections from the manuscript (include quotes in quotation marks "like this" to indicate direct quotes from your manuscript), or elaborate on this item by providing additional information not in the ms, or briefly explain why the item is not applicable/relevant for your study

Meine Antwort

DISCUSSION

22) Interpretation consistent with results, balancing benefits and harms, and considering other relevant evidence

NPT: In addition, take into account the choice of the comparator, lack of or partial blinding, and unequal expertise of care providers or centers in each group

22-i) Restate study questions and summarize the answers suggested by the data, starting with primary outcomes and process outcomes (use)

Restate study questions and summarize the answers suggested by the data, starting with primary outcomes and process outcomes (use).

subitem not at all important

1 ○

2 ○

3 ◉

4 ○

5 ○

essential

Auswahl löschen

Does your paper address subitem 22-i? *

Copy and paste relevant sections from the manuscript (include quotes in quotation marks "like this" to indicate direct quotes from your manuscript), or elaborate on this item by providing additional information not in the ms, or briefly explain why the item is not applicable/relevant for your study

"Furthermore, across conditions, participants showed a significant increase in the frequency of performing health-promoting behaviors after being confronted with the intervention materials. However, this effect was only driven by the delayed intervention group, whereas the early intervention group did not increase in health-promoting behaviors."

22-ii) Highlight unanswered new questions, suggest future research

Highlight unanswered new questions, suggest future research.

subitem not at all important

1 ○

2 ○

3 ●

4 ○

5 ○

essential

Auswahl löschen

Does your paper address subitem 22-ii?

Copy and paste relevant sections from the manuscript (include quotes in quotation marks "like this" to indicate direct quotes from your manuscript), or elaborate on this item by providing additional information not in the ms, or briefly explain why the item is not applicable/relevant for your study

Nevertheless, further steps are needed to test whether the present findings replicate and can be generalized. First, direct replications could test whether the effectiveness of an incremental theories intervention is indeed time-sensitive, as demonstrated in this study. In addition, future studies should also focus on testing the longevity of the intervention effect. Investigating whether the increase in incremental theories can be sustained over longer periods and whether this increase translates into sustained improvements in health behaviors is essential. Therefore, studies with increased follow-up periods are necessary to test the longevity of the intervention effect and the respective impact on health behaviors. Next, conceptual replications could investigate what modes of delivering an implicit theories intervention are most effective and for whom. For example, the effectiveness might be higher when the changeability of health is emphasized several times over an extended course of time. A useful tool in this respect could be an app that tracks and visualizes changes in one’s health behavior over time. Finally, the generalizability to different populations and different health behavior measures needs to be ensured. Research on implicit theories of health would also benefit greatly from examining the antecedents and determinants that lead to adopting an incremental versus entity theory [7]. Auster-Gussman and Rothman [24] found that incremental theories of body weight are more common among young and White individuals with a higher level of income and education. These variables, as well as one’s medical history or that of close others, should also play a significant role in the formation of implicit theories [7]. This study shows that even a rather young and educated sample with high self-reported health and high incremental theories at baseline benefits from an incremental theories intervention. Effects might be stronger when studying population groups with higher entity theories of health."

20) Trial limitations, addressing sources of potential bias, imprecision, and, if relevant, multiplicity of analyses

20-i) Typical limitations in ehealth trials

Typical limitations in ehealth trials: Participants in ehealth trials are rarely blinded. Ehealth trials often look at a multiplicity of outcomes, increasing risk for a Type I error. Discuss biases due to non-use of the intervention/usability issues, biases through informed consent procedures, unexpected events.

subitem not at all important

1 ○

2 ◉

3 ○

4 ○

5 ○

essential

Auswahl löschen

Does your paper address subitem 20-i? *

Copy and paste relevant sections from the manuscript (include quotes in quotation marks "like this" to indicate direct quotes from your manuscript), or elaborate on this item by providing additional information not in the ms, or briefly explain why the item is not applicable/relevant for your study

"Future trials should include a no-treatment control group that does not receive intervention materials. This approach makes it possible to determine whether the pre-post difference in behavior in the delayed intervention group appeared because of the intervention materials or because of the combination of the intervention materials with daily diaries."

21) Generalisability (external validity, applicability) of the trial findings

NPT: External validity of the trial findings according to the intervention, comparators, patients, and care providers or centers involved in the trial

21-i) Generalizability to other populations

Generalizability to other populations: In particular, discuss generalizability to a general Internet population, outside of a RCT setting, and general patient population, including applicability of the study results for other organizations

subitem not at all important

1 ○

2 ○

3 ◉

4 ○

5 ○

essential

Auswahl löschen

Does your paper address subitem 21-i?

Copy and paste relevant sections from the manuscript (include quotes in quotation marks "like this" to indicate direct quotes from your manuscript), or elaborate on this item by providing additional information not in the ms, or briefly explain why the item is not applicable/relevant for your study

"Regarding the generalizability of findings, it is essential to note that the surveyed sample differs from the general population, especially regarding age, educational level, and student proportion. Participation in the study required owning a smartphone with internet access, and recruitment was realized via social media and mailing lists. This has limited the studies' accessibility for individuals of older age."
"Finally, the generalizability to different populations and different health behavior measures needs to be ensured."

21-ii) Discuss if there were elements in the RCT that would be different in a routine application setting

Discuss if there were elements in the RCT that would be different in a routine application setting (e.g., prompts/reminders, more human involvement, training sessions or other co-interventions) and what impact the omission of these elements could have on use, adoption, or outcomes if the intervention is applied outside of a RCT setting.

subitem not at all important

1 (selected)

2

3

4

5

essential

Auswahl löschen

Does your paper address subitem 21-ii?

Copy and paste relevant sections from the manuscript (include quotes in quotation marks "like this" to indicate direct quotes from your manuscript), or elaborate on this item by providing additional information not in the ms, or briefly explain why the item is not applicable/relevant for your study

Meine Antwort

OTHER INFORMATION

23) Registration number and name of trial registry

Does your paper address CONSORT subitem 23? *

Copy and paste relevant sections from the manuscript (include quotes in quotation marks "like this" to indicate direct quotes from your manuscript), or elaborate on this item by providing additional information not in the ms, or briefly explain why the item is not applicable/relevant for your study

It was registered as a randomized trial in the German Clinical Trials Register (trial number DRKS00017379)

24) Where the full trial protocol can be accessed, if available

Does your paper address CONSORT subitem 24? *

Cite a Multimedia Appendix, other reference, or copy and paste relevant sections from the manuscript (include quotes in quotation marks "like this" to indicate direct quotes from your manuscript), or elaborate on this item by providing additional information not in the ms, or briefly explain why the item is not applicable/relevant for your study

https://drks.de/search/en/trial/DRKS00017379

25) Sources of funding and other support (such as supply of drugs), role of funders

Does your paper address CONSORT subitem 25? *

Copy and paste relevant sections from the manuscript (include quotes in quotation marks "like this" to indicate direct quotes from your manuscript), or elaborate on this item by providing additional information not in the ms, or briefly explain why the item is not applicable/relevant for your study

"This work was supported by an advanced postdoctoral grant from the University of Cologne awarded to SD. Open access funding was provided by the University of Vienna"

X27) Conflicts of Interest (not a CONSORT item)

X27-i) State the relation of the study team towards the system being evaluated

In addition to the usual declaration of interests (financial or otherwise), also state the relation of the study team towards the system being evaluated, i.e., state if the authors/evaluators are distinct from or identical with the developers/sponsors of the intervention.

subitem not at all important

1 ○

2 ◉

3 ○

4 ○

5 ○

essential

Auswahl löschen

Does your paper address subitem X27-i?

Copy and paste relevant sections from the manuscript (include quotes in quotation marks "like this" to indicate direct quotes from your manuscript), or elaborate on this item by providing additional information not in the ms, or briefly explain why the item is not applicable/relevant for your study

None conflicts of interest declared

About the CONSORT EHEALTH checklist

As a result of using this checklist, did you make changes in your manuscript? *

○ yes, major changes

○ yes, minor changes

◉ no

What were the most important changes you made as a result of using this checklist?

Meine Antwort

How much time did you spend on going through the checklist INCLUDING making changes in your manuscript *

It took approximately 90 MInutes

As a result of using this checklist, do you think your manuscript has improved? *

- ○ yes
- ◉ no
- ○ Sonstiges:

Would you like to become involved in the CONSORT EHEALTH group?

This would involve for example becoming involved in participating in a workshop and writing an "Explanation and Elaboration" document

- ○ yes
- ◉ no
- ○ Sonstiges:

Auswahl löschen

Any other comments or questions on CONSORT EHEALTH

Meine Antwort

STOP - Save this form as PDF before you click submit

To generate a record that you filled in this form, we recommend to generate a PDF of this page (on a Mac, simply select "print" and then select "print as PDF") before you submit it.

When you submit your (revised) paper to JMIR, please upload the PDF as supplementary file.

Don't worry if some text in the textboxes is cut off, as we still have the complete information in our database. Thank you!

Final step: Click submit !

Click submit so we have your answers in our database!

Senden Alle Eingaben löschen

Geben Sie niemals Passwörter über Google Formulare weiter.

Dieser Inhalt wurde nicht von Google erstellt und wird von Google auch nicht unterstützt. Missbrauch melden - Nutzungsbedingungen - Datenschutzerklärung

Google Formulare
